# CRUSH—Cleavage Rules Using SMIRKS Heuristics: an enhanced molecular fragmentation algorithm

**DOI:** 10.1186/s13321-026-01239-w

**Published:** 2026-06-13

**Authors:** Edgar López-López, José L. Medina-Franco, Filip Miljković

**Affiliations:** 1 DIFACQUIM Research Group, Department of Pharmacy, School of Chemistry, Universidad Nacional Autónoma de México, Avenida Universidad 3000, 04510 Mexico City, Mexico; 2 Department of Pharmaceutical Biosciences, Uppsala University, Box 591, 75124 Uppsala, Sweden; 3 Biopharma Chemistry, Discovery Sciences, BioPharmaceuticals R&D, AstraZeneca, Pepparedsleden 1, 43183 Mölndal, Sweden

**Keywords:** Chemical space, Chemical libraries, Compound design, Fragment libraries, Molecular fragmentation, Open source

## Abstract

Molecular fragmentation methods are central to fragment-based discovery, virtual screening, and *de novo* molecular design, yet most existing approaches remain constrained by classical retrosynthetic rules that limit the scope and chemical space exploration beyond medicinal chemistry applications. Here we introduce CRUSH (Cleavage Rules Using SMIRKS Heuristics), an enhanced, chemistry-aware fragmentation algorithm that extends traditional retrosynthetic bond disconnection and building-block generation strategies to enable fine-grained and chemically meaningful molecular deconstruction. CRUSH adopts a flexible bond cleavage strategy in which a curated set of SMIRKS-based heuristics is applied exhaustively across all eligible bonds at each fragmentation step, enabling systematic exploration of multiple alternative disconnection pathways rather than committing to the first applicable retrosynthetic rule. Using a unified benchmarking framework across five chemically diverse datasets, including drug-like molecules, natural products, peptides, macrocycles, and alimentary compounds, we demonstrate that CRUSH consistently increases fragment yield and substantially expands chemical space coverage compared with the well-established methods. Notably, CRUSH-derived fragments access exclusive regions of chemical space that remain unexplored by existing approaches, a result that is consistent across both low- and high-resolution molecular fingerprint representations. Structurally, CRUSH generates smaller and less complex fragments, a profile particularly advantageous for fragment-based and diversity-driven workflows. Collectively, these results establish CRUSH as a robust and versatile molecular fragmentation framework that complements, and in exploratory contexts surpasses, current methods, providing a strong foundation for fragment-based chemoinformatics applications across multiple chemistry domains. As part of this work, implementation of the CRUSH fragmentation algorithm is made freely available at https://github.com/EdgL2/CRUSH.

**Scientific contribution**

This study introduces CRUSH, a chemistry-aware molecular fragmentation method that extends beyond traditional medicinal chemistry retrosynthetic rules to enable fine-grained and chemically meaningful bond disconnections. CRUSH consistently increases fragment yield and uniquely expands chemical space coverage across diverse datasets and molecular representations, generating smaller and less complex fragments well suited for fragment-based, exploratory, and *de novo* design workflows.

**Supplementary Information:**

The online version contains supplementary material available at 10.1186/s13321-026-01239-w.

## Introduction

Molecular fragmentation plays a central role in chemistry, enabling the systematic decomposition of complex molecules into smaller, chemically interpretable substructures. Such approaches underpin fragment-based drug design, chemical space exploration, library design, and structure–activity relationship (SAR) analysis, where the identification and recombination of meaningful fragments—often conceived as building blocks that are commercially available for purchase and use in organic synthesis—facilitate both rational design and large-scale computational screening [1, 2]. By reducing molecular complexity while preserving chemical identity, fragmentation methods provide a practical bridge between molecular structure and fragment-based data-driven discovery workflows.

In parallel with these methodological advances, chemical databases have expanded rapidly in both size and diversity, encompassing approved drugs, bioactive compounds, natural products, peptides, and complex macrocycles [3–5], among others. This growth has intensified the need for scalable, chemically coherent, and comprehensive fragmentation strategies that can extract informative substructures across heterogeneous chemical spaces. Effective fragmentation is increasingly critical not only for database organization and diversity analysis but also for enabling artificial intelligence-based approaches, where chemically decomposed representations can enhance data density, interpretability, and model performance [6].

Rule-based fragmentation algorithms have emerged as a dominant strategy to address these needs by decomposing molecules according to predefined chemically or synthetically motivated bond disconnections. Among the most widely adopted methods are RECAP (Retrosynthetic Combinatorial Analysis Procedure) [7] and BRICS (Breaking of Retrosynthetically Interesting Chemical Substructures) [8], both of which apply expert-derived transformation rules to systematically segment molecular graphs into chemically meaningful fragments. In parallel, there has been a growing interest in software frameworks that not only encapsulate individual rule sets but also support the development, visualization, and comparative evaluation of fragmentation strategies. One such framework is MORTAR (MOlecule fRagmenTAtion fRamework) [9], an open-source application that integrates multiple fragmentation and substructure analysis algorithms within a unified graphical user interface. This is achieved by lowering the barrier to applying and comparing rule-based fragmentation techniques and by providing rich downstream analysis functions.

RECAP is grounded in classical retrosynthetic logic, defining fragmentation rules based on commonly formed or broken bonds in organic synthesis [7]. BRICS, in contrast, emphasizes the generation of synthetically tractable building blocks, focusing on bond types that facilitate recombination into chemically feasible molecules [8]. While both approaches have proven valuable, they reflect different priorities in terms of chemical representation and downstream applicability. Despite their widespread use, systematic and large-scale comparisons of fragmentation algorithms across diverse chemical databases remain limited. Several studies focus on isolated use cases, specific datasets, or individual algorithms, leaving open questions regarding how fragmentation logic influences fragment diversity, chemical space coverage, and suitability for exploratory or data-driven applications [10–13].

In this work we introduce CRUSH, a molecular fragmentation algorithm designed to extend classical retrosynthetic and building-block-oriented approaches through a fine-grained, chemistry-aware bond disconnection strategy. CRUSH aims to generate chemically meaningful fragments with increased diversity and broader chemical space coverage. As part of this work, we discuss a comparison of CRUSH with RECAP and BRICS algorithms across multiple chemically diverse datasets, evaluating their performance, fragment characteristics, and suitability for exploratory and fragment-based workflows.

## Methods

### Datasets

Representative and chemically diverse datasets considered in this study included small-molecule drugs (ChemDiv) [14], natural products (Latin American Natural Product Database—LANaPDB v.2) [15], antimicrobial peptides (anti-MRSA) [16], macrocycles (Macrocycle-DB) [17], and food-related chemicals (FooDB) [18]. Compound data sets were analyzed to characterize and compare the chemical space coverage of fragments obtained using different fragmentation algorithms (*vide infra*). A summary of each dataset is provided below.

The inhibitors subset from ChemDiv comprises 41,068 small molecules with desirable drug-like properties, including approved drugs, compounds considered for drug repurposing, and molecules that have progressed to clinical evaluation [14]. LANaPDB v2 includes 13,578 natural products from Latin America and is characterized by a high degree of structural diversity and broader chemical space coverage relative to approved drugs [15]. The anti–methicillin-resistant *Staphylococcus aureus* (anti-MRSA) dataset consists of 223 peptides featuring canonical and non-canonical amino acids, as well as post-translational and synthetic modifications, that are characterized by diverse linear and cyclic architectures [16]. Macrocycle-DB contains 46,089 compounds classified as cyclic peptides, synthetic macrocycles, natural products, macrolides, porphyrins, and macrocyclic polyenes [17]. Finally, FooDB comprises 70,477 compounds, including macronutrients, micronutrients, and food-associated chemicals related to flavor, color, taste, texture, and aroma [18]. For all chemical datasets, SMILES strings were parsed and sanitized using RDKit and converted to canonical form prior to fragmentation [19].

### CRUSH fragmentation rules

CRUSH (Cleavage Rules Using SMIRKS Heuristics) is introduced here as a curated rule set designed to formalize chemically meaningful fragmentations of molecules belonging to a diverse, wide range of chemicals of different origins. Rather than treating molecular fragmentation as a purely algorithmic operation, CRUSH encodes SMIRKS rules [20] that explicitly capture bond types, chemical environments, and functional group contexts commonly encountered in diverse chemical datasets. Each rule represents a chemically interpretable transformation, defining how specific covalent linkages—such as amides, esters, heteroatom–carbon bonds, and aromatic connections—are systematically uncoupled into well-defined substructures with preserved attachment vectors. By operating at the level of reaction logic rather than generic bond breaking, CRUSH enables consistent, reproducible, and chemically grounded decomposition of molecular architectures, with the potential of facilitating downstream tasks such as scaffold analysis, linker prioritization, and exploration of structure–property associations. The CRUSH rule set was designed to generalize and extend concepts originally introduced in the RECAP and BRICS fragmentation frameworks [7, 8], while substantially increasing the diversity of bond types and chemical contexts considered.

Several CRUSH rules target functional group disconnections common in medicinal chemistry that are conceptually analogous to those implemented in RECAP and BRICS, including amides, esters, ureas, carbamates, amines, ethers, and sulfonamides [7, 8]. These rules preserve linker motifs commonly used in medicinal chemistry and fragment-based design. However, in contrast to the more conservative scope of RECAP and BRICS, CRUSH incorporates a broader range of bond environments and explicitly differentiates between aliphatic, aromatic, and heterocyclic contexts.

The CRUSH rule set includes fragmentation patterns for a wide spectrum of structural motifs, comprising carbon–heteroatom bonds (e.g., aryl C–O, aryl C–N, aryl C–S, aliphatic C–heteroatom), carbon–carbon bonds (e.g., aliphatic C–C, olefinic, alkyne, aryl–alkyl C–C, and aryl–vinyl C–C linkages), and heterocycle-associated disconnections (e.g., lactams, aromatic heterocycles, etc.). In addition, rules addressing sulfur-containing functionalities (e.g., thioethers, disulfides, sulfonate esters, and acylsulfonamides) and motifs arising from modern synthetic strategies were explicitly incorporated. The full list of SMIRKS rules of CRUSH and their descriptions is available in Table 1. In total, the CRUSH fragmentation scheme introduced herein comprises 33 distinct SMIRKS-based rules, each of which was capped with dummy-type atoms to facilitate tracking of each fragmentation rule across multiple datasets.

**Table 1:** List of SMIRKS rules of the CRUSH fragmentation algorithm

Rule	Description	Examples of compounds fragmented	SMIRKS
Acyclic carbonyl-N cleavage	Cleaves a non-ring carbonyl–nitrogen single bond, generating a carbonyl-containing fragment and a nitrogen-containing fragment	Any containing acyclic carbonyl–nitrogen (C(=O)–N) bond, including amides, peptides, ureas, carbamates, hydrazides, and related carbonyl–nitrogen functionalities	[#6:1]!@[C!R:2](=[O:3])!@[N:4] >> [*:1][*:2](=[*:3]).[*:4]
Ester	Breaks a non-ring ester linkage between a carbonyl carbon and an alkoxy oxygen, yielding a carbonyl fragment and an alkoxy-containing fragment	Any acyclic carboxylic ester with the motif R–C(=O)–O–R′, including aliphatic and aromatic esters	[#6:1]!@[C!R:2](=[O:3])!@[O!R:4][#6:5] >> [*:1][*:2](=[*:3]).[*:4][*:5]
Tertiary/quaternary aliphatic amine C–N cleavage	Cleaves an aliphatic N–C bond in tertiary or quaternary amines, generating an amine-containing fragment and a separated sp^3^ carbon fragment	Any containing a tertiary amine or quaternary ammonium center (NX3 or N⁺X4) bound to three sp^3^ carbon substituents (CX4), encompassing trialkylamines, substituted cyclic amines, and quaternary ammonium derivatives	[NX3,N+X4:1]([CX4:2])([CX4:3])[CX4:4] >> [*:1]([*:2])([*:3]).[*:4]
Ring tertiary amine substituent disconnect	Disconnects an exocyclic N–C(sp^3^) bond of a ring-bound tertiary amine, producing a cyclic tertiary amine fragment and an aliphatic substituent fragment	Any containing a ring-bound tertiary amine (NX3) in which at least one substituent is an exocyclic sp^3^ carbon (CX4), such that an N–C(sp^3^) bond external to the ring can be cleaved. This includes substituted cyclic tertiary amines bearing alkyl side chains	[N;D3;R:1]!@[C;!$(C=*):2] >> [*:1].[*:2]
Urea	Cleaves both C–N bonds of a non-ring urea functional group, generating a carbonyl-containing fragment and two amine-containing fragments	Any containing non-ring urea linkage (N–C(=O)–N) subject to double C–N bond fragmentation	[N:1]!@[C!R:2](=[O:3])!@[N:4] >> [*:1][*:2](=[*:3]).[*:4]
Ether	Breaks a non-ring C–O ether bond, splitting the molecule into a C(H)–C–O fragment and a C–C(H) fragment	Any containing an acyclic ether (C–O–C) linkage, including simple dialkyl ethers and related non-cyclic ether functionalities	[#6,#1:1][#6:2]!@[O!R:3]!@[#6:4][#6,#1:5] >> [*:1][*:2][*:3].[*:4][*:5]
Hetero-substituted alkyl C–X cleavage	Cleaves a single bond between an aliphatic ring carbon singly bounded to a heteroatom (N, O, or S) and any adjacent atom within a ring system, producing two disconnected fragments	Any containing an aliphatic carbon bonded via a single bond to a heteroatom (N, O, or S), encompassing several functional classes such as aliphatic ethers (C–O–C), aliphatic amines (C–N), and thioethers (C–S)	[C;$(C(-;@[C,N,O,S])-;@[N,O,S]):1]-[*:2] >> [*:1].[*:2]
Olefin	Cleaves a non-ring carbon–carbon double bond, yielding two disconnected fragments with defined attachment points	Any containing an aliphatic, non-ring C=C bond, characteristic of acyclic alkenes and alkene-containing chains	[C:1]!@=[C:2] >> [*:1].[*:2]
Quaternary nitrogen	Breaks the bond between a quaternary ammonium nitrogen and an attached carbon, generating two disconnected fragments while preserving the positive charge on the nitrogen	Any containing a quaternary ammonium nitrogen bonded to a carbon through a non-ring bond, characteristic of quaternary ammonium salts and charged alkylammonium groups	[N+X4&H0:1]!@[C:2] >> [*:1].[*:2]
Aromatic nitrogen–aliphatic carbon	Disconnects an aromatic nitrogen atom from an aliphatic carbon, yielding an aromatic heterocycle fragment and an aliphatic carbon fragment	Any containing an aromatic heterocycle in which an aromatic nitrogen is bound to an aliphatic carbon (n–C non-ring bond), as found in N-alkylated aromatic heterocycles such as N-substituted pyridinium, imidazolium, or related aromatic nitrogen systems	[n,n+:1]!@[C:2] >> [*:1].[*:2]
Lactam nitrogen–aliphatic carbon	Cleaves the N–C bond in a lactam where the nitrogen is attached to an aliphatic carbon, preserving the lactam carbonyl within the cyclic fragment	Any containing N-alkylated cyclic amides (lactams), including monocyclic and fused lactam systems	[NR:1](@[CR;!$(C=[O,N]):2])(!@[C:3])@[CR:4]=[O:5] >> [*:1]([*:2])[*:4]=[*:5].[*:3]
Aromatic carbon–aromatic carbon	Breaks a biaryl C–C bond, generating two independent aromatic fragments	Any containing two aromatic rings connected by a non-ring C–C bond, including both symmetric and asymmetric biaryl frameworks, including substituted biphenyls, naphthyl–phenyl compounds, and heteroaryl–aryl systems	[c:1]!@[c:2] >> [*:1].[*:2]
Sulphonamide	Cleaves a sulfonamide N–S bond, yielding a sulfonyl fragment and an amine-containing fragment	Any containing a sulfonamide N–S bond, including both simple and substituted, such as alkyl- or aryl-sulfonamides	[S!R:1](=[O:2])(=[O:3])!@[N:4]!@[#6;!$(C = [O,N]):5] >> [*:1](=[*:2])(=[*:3]).[*:4][*:5]
Aromatic carbon–aliphatic carbon	Disconnects an aromatic carbon from an sp^3^ aliphatic carbon that is not bonded to heteroatoms, producing separate aromatic and aliphatic fragments	Any containing a non-aromatic bond between an aromatic C and a sp^3^ aliphatic C not directly attached to heteroatoms, including both simple and substituted systems, such as benzyl, phenethyl, and related aryl–alkyl frameworks	[c:1]!@[CX4;!$(C~[!#1&!#6]):2] >> [*:1].[*:2]
Aromatic carbon–aliphatic amine	Cleaves an aromatic carbon–nitrogen bond linking an aryl ring to an aliphatic amine	Any containing a C–N bond linking an aryl ring to an aliphatic amine, including both simple and substituted examples, such as anilines, N-alkyl anilines, and related aryl–amine compounds	[c:1]!@[N:2] >> [*:1].[*:2]
Alkyne	Breaks an acyclic carbon–carbon triple bond, producing two alkyne-derived fragments	Any containing a C–C triple bond, including both simple and substituted alkynes	[C:1]!@#[C:2] >> [*:1].[*:2]
Thioether	Cleaves a non-ring thioether C–S bond, generating separate carbon and sulfur-containing fragments	Any containing a non-ring thioether C–S–C linkage, including both simple and substituted, such as linear or branched thioethers and related sulfur-containing compounds	[#6;!R:1]!@[S;X2:2]!@[#6;!R:3] >> [*:1].[*:2][*:3]
Carbamate_1	Breaks a carbamate carbonyl–oxygen bond, producing an alcohol fragment and a carbamoyl (amide-like) fragment	Any containing a non-ring carbamate N–C(=O)–O linkage, including both simple and substituted, such as alkyl- or aryl-carbamates and related amine–carbonyl–oxygen systems	[O:1]!@[C!R:2](=[O:3])!@[N:4] >> [*:1][*:2](=[*:3]).[*:4]
Carbamate_2	Cleaves a non-ring carbamate into three fragments: alkoxy, carbonyl, and amine components	Any containing a non-ring O–C(=O)–N linkage, including both simple and substituted examples, such as alkyl- or aryl-carbamates, and related oxygen–carbon–nitrogen systems, excluding ureas	[O;!R:1]!@[C;!R:2](=[O:3])!@[N;!R;!$([N]-[C](= O)-[N]):4] >> [*:1].[*:2](=[*:3]).[*:4]
Acyl-sulphamide	Breaks an acylsulphamide linkage, yielding a sulphamide-containing fragment and an acyl fragment	Any containing a non-ring N–C(=O) linkage within a sulfonamide, including both simple and substituted examples, such as alkyl- or aryl-acylsulfonamides and related sulfonamide–acyl systems	[#6:1][S!R:2](=[O:3])(=[O:4])!@[N:5]!@[C!R:6](=[O:7]) >> [*:1][*:2](=[*:3])(=[*:4])[*:5].[*:6](=[*:7])
Sulfacylation-O	Cleaves an O–S bond in an aryl sulfonate ester, generating a sulphonate fragment and an aryloxy fragment	Any containing a non-ring O–S linkage within an aryl sulfonate ester, including both simple and substituted examples, such as aryl-sulfonates and related aryloxy–sulfonate systems	[S!R:1](=[O:2])(=[O:3])!@[O:4]!@[c:5] >> [*:1](=[*:2])(=[*:3]).[*:4][*:5]
Aryl-C–N cleavage	Breaks a single C–N bond between an aromatic carbon and an aromatic nitrogen, generating two fragments; it only affects exocyclic C–N linkages	Any containing a non-ring C–N bond, including both simple and substituted examples, such as aliphatic amines, N-alkyl anilines, and related aryl–amine or alkyl–amine systems	[c:1]!@[n:2] >> [*:1].[*:2]
Inter-Aryl-N–N cleavage	Cleavage of a single N–N bond directly connecting two heteroaromatic rings. The rule generates two independent heteroaromatic fragments and is intended for scaffold decomposition	Any system containing two heteroaromatic rings directly connected by a single N–N bond, where each N belongs to an aromatic system. Representative examples include azole–azole, azole–azine, and azine–azine scaffolds Excluding azo (N=N) bonds, aliphatic hydrazines (non-aromatic N–N), and intra-ring N–N bonds	[n:1]!@[n:2] >> [*:1].[*:2]
Ar–alkyl cleavage	Cleaves a single bond between an aromatic carbon and a non-ring aliphatic carbon, generating two disconnected fragments by replacing the bond with generic attachment points	Any containing a single bond between an aromatic C and a non-ring aliphatic C, including both simple and substituted examples, such as benzyl derivatives, arylalkanes, and related aromatic–aliphatic systems	[c:1]-[C;!R:2] >> [*:1].[*:2]
C–C break (aliphatic)	Cleaves a non-ring, non-carbonyl aliphatic carbon–carbon single bond	Any containing a non-ring, non-carbonyl aliphatic C–C single bond, including both simple and substituted examples, such as linear or branched alkanes, alkyl chains, and related saturated aliphatic systems	[C;!R;!$(C=[O,N]):1]!@[C;!R;!$(C=[O,N]):2] >> [*:1].[*:2]
C–hetero (aliphatic)	Breaks a single bond between an aliphatic carbon and a non-carbon heteroatom	Any containing a non-ring aliphatic C–X single bond, where X is a non-carbon heteroatom, including both simple and substituted examples, such as alcohols, amines, thiols, alkyl halides, and related compounds	[C;!R:1]!@[!#6;!R:2] >> [*:1].[*:2]
Sulfonate ester cleavage	Cleaves the O–C bond of a sulfonate ester, yielding a sulfonic acid fragment and an alkyl fragment	Any containing a sulfonate ester O–C linkage, including both simple and substituted examples, such as alkyl- or aryl-sulfonates and related sulfonate–alkyl systems	[S!R:1](=[O:2])(=[O:3])!@[O:4]!@[C:5] >> [*:1](=[*:2])(=[*:3])[*:4].[*:5]
Disulfide bond cleavage	Breaks a disulfide bond, producing two thiol-derived fragments	Any containing a disulfide (S–S) linkage, including both simple and substituted examples	[S:1]-[S:2] >> [*:1].[*:2]
Ar-vinyl-core cleavage	Cleaves the bond between an aromatic ring and a vinyl linkage, retaining the C=C alkene core while leaving substituents on the alkene carbons unconstrained	Any containing a bond between an aromatic C and a vinyl C, including both simple and substituted examples, such as styrenes, vinyl-substituted aromatics, and related aryl–alkene systems	[c:1]-[C:2]=[C:3] >> [*:1].[*:2]=[*:3]
Ar–O cleavage	Breaks an aromatic carbon–oxygen single bond, generating separate aromatic and oxygen-containing fragments	Any containing a single bond between an aromatic carbon and an oxygen atom, including both simple and substituted examples, such as aromatic ethers, phenols, and related aryl–oxygen systems	[c:1]-[O:2] >> [*:1].[*:2]
Ar–N exocyclic cleavage	Fragments molecules by breaking a single bond between an aromatic carbon and a nitrogen atom, producing separate carbon and nitrogen fragments. The nitrogen may be part of an exocyclic or open-chain group	Any containing a single bond between an aromatic carbon and a nitrogen atom, including both simple and substituted examples, such as anilines, N-alkyl anilines, aromatic amides (where the nitrogen is exocyclic), and related aryl–nitrogen systems	[c:1]-[N:2] >> [*:1].[*:2]
Ar–S exocyclic cleavage	Fragments an exocyclic aryl–sulfur bond, generating separate aromatic and sulfur-containing moieties	Any containing a non-ring aromatic–sulfur bond, including both simple and substituted examples, such as aryl thioethers, aryl–sulfenyl derivatives, and related sulfur-containing aromatic systems	[c:1]-[S:2] >> [*:1].[*:2]
Ar–sp–sp C cleavage	Disconnects an aromatic ring from an alkynyl substituent	Any containing a non-ring aromatic–alkynyl linkage, including both simple and substituted examples, such as aryl-alkynes, aryl-acetylenes, and related aromatic systems bearing terminal or internal alkynyl substituents	[c:1]-[C:2]#[C:3] >> [*:1].[*:2]#[*:3]

Unlike RECAP and BRICS, which largely avoid cleavage of carbon–carbon bonds, CRUSH explicitly enables C–C disconnections. To reduce chemically implausible fragmentations, several CRUSH rules were defined with explicit constraints on ring membership, substitution patterns, and local chemical environments.

### Molecular fragmentation

Molecular fragmentation was performed using a parallel, streaming workflow implemented in Python. Two established rule-based fragmentation schemes were evaluated: RECAP and BRICS, which were compared with the CRUSH fragmentation algorithm proposed in this study, each defined by a curated and explicitly specified set of SMIRKS transformation rules [20]. All reaction rules were precompiled into RDKit ChemicalReaction objects to ensure computational efficiency and reproducibility.

Input molecules were provided as Simplified Molecular Input Line Entry System (SMILES) [21] standardized strings within a comma-separated values (CSV) file, containing a unique molecular identifier and a SMILES column. To enable scalable processing of large chemical libraries, the input file was read in chunks, thereby limiting peak memory usage. Fragmentation tasks were distributed across multiple CPU (central processing unit) cores using Python’s multiprocessing module, reserving one core for the main process to manage input/output operations. Each worker process independently applied the fragmentation workflow to individual molecules, while a single writer process ensured ordered and synchronized output of molecular metadata and binary fingerprints.

Fragmentation was performed using a single, unified recursive script applied identically across all rule sets to ensure a fair and controlled comparison (i.e., RECAP and BRICS rules were executed within the same framework as CRUSH). For each molecule and rule set (RECAP, BRICS, and CRUSH), all applicable SMIRKS transformations were exhaustively evaluated under identical computational constraints. The applied rules were designed to generate complementary fragments such that, when considered collectively, the original molecular structure can be fully reconstructed from the resulting fragment set, preserving chemical consistency across fragmentation strategies. Fragmentation was subject to the following constraints: (1) Minimum fragment size: fragments containing fewer than three heavy atoms were discarded; (2) Ring protection: atoms participating in fused ring systems, and optionally their neighboring atoms, were protected from bond cleavage; (3) Conservative mode: reactions affecting protected atoms were excluded entirely to preserve core ring integrity.

At each recursion step, reaction products were converted to canonical SMILES representations, and fragmentation proceeded iteratively until no further valid transformations were possible. Among all explored pathways, the fragmentation route that maximized the total number of retained fragments was selected for downstream analysis.

### Fingerprint calculation

For each resulting fragment, molecular fingerprints (ECFP4 2048-bit and MACCS keys 166-bit) were computed [22, 23]. Fingerprints were converted into NumPy arrays, packed into byte representations using bitwise encoding, and appended sequentially to a binary file. This approach minimized disk usage and enabled efficient downstream batch processing. Simultaneously, fragment-level metadata, including parent molecule identifier, input SMILES, fragment SMILES, applied reaction rule, and fragmentation mode, were buffered and periodically written to a CSV file in batches to reduce I/O overhead.

### Comparative analysis of different rule-based fragment set algorithms

To quantitatively compare the overlap and uniqueness of molecular fragments generated by different fragmentation algorithms, a data-driven analysis was implemented using a custom Python workflow executed in Google Colab (see Data availability section).

For each fragmentation method, all unique identifiers were extracted and subsequently reduced to unique entries, which were stored as Python sets. This set-based representation ensures that duplicate identifiers arising from the same fragmentation method are collapsed and counted only once. As a result, the analysis focuses exclusively on the unique fragment space associated with each algorithm, thereby minimizing potential biases arising from differences in fragment counts across methods.

Standard comparison operations were used to organize the identified fragments into groups based on whether they were unique to a single fragmentation method, shared by two methods, or common to all three. By examining these overlaps and differences, we were able to quantify how the methods related to each other in a clear and reproducible manner, while highlighting both shared fragment space and method-specific contributions. The total number of unique identifiers across all methods was defined as the union of the three sets and used as the reference for relative frequency calculations. A three-set Venn diagram was generated using the ‘matplotlib-venn’ library to visually summarize the relationships between the fragment spaces produced by RECAP, BRICS, and CRUSH.

### Fragment space construction

To visualize the chemical space of large fragment collections, molecular fingerprints were transformed into low-dimensional representations using a two-step approach. First, different numbers of principal components were evaluated in order to retain as much of the original variance as possible from the fingerprint data. The reduced representations were then projected into two dimensions using Uniform Manifold Approximation and Projection (UMAP) method, enabling intuitive visualization of fragment distributions and relationships [24]. UMAP was applied with a Euclidean distance metric, random initialization, and fixed random seeds to ensure reproducibility. The resulting two-dimensional embeddings captured the global and local structure of the fragment chemical space. UMAP coordinates were merged with the fragment metadata table. The combined dataset, containing fragment identities, fragmentation provenance, and low-dimensional coordinates, was exported as a final CSV file suitable for visualization and further analysis (see Data availability section).

### Chemical space analysis of fragmentation methods

A comprehensive post-fragmentation analysis workflow was implemented in Google Colab to evaluate the structural relationship, distributional properties, and chemical space coverage of fragments generated by different fragmentation strategies, namely CRUSH, BRICS, and RECAP. The methodology integrates molecular similarity calculations, statistical visualization, frequency analysis, and chemical space mapping using spatial overlap metrics.

Fragmentation results were provided as a CSV file, which was uploaded interactively into the Google Colab environment. The dataset was loaded using the pandas library, with the following columns requested as input: Original_SMILES (SMILES string of the compound), Fragment_SMILES (SMILES string of one of the corresponding fragments), Mode (fragmentation method), Rule (specific fragmentation rule), and UMAP_1 and UMAP_2 (precomputed low-dimensional embeddings).

For each compound–fragment pair, molecular similarity was quantified using the Tanimoto coefficient [25] computed over ECFP4 fingerprint (2048 bits), as implemented in RDKit [19]. The resulting similarity values were stored as a new column (Similarity) in the dataset, enabling downstream statistical and comparative analyses across fragmentation methods.

To compare how closely the fragments resembled their parent molecules across different fragmentation strategies, kernel density estimation (KDE) curves were generated for each Mode. As each fragmentation method generated an immense number of compound-fragment relationships, KDE representation helped to aggregate individual data points and display their frequency that is not typically observed in standard scatter plot visualizations of chemical similarity distributions. KDE plots were computed separately for each method using consistent bandwidth and transparency parameters to facilitate visual comparison.

Chemical space coverage and overlap were quantified using a grid-based occupancy approach. The UMAP space was discretized into a fixed-resolution two-dimensional grid, and binary occupancy matrices were constructed globally and per fragmentation method [26]. Pairwise overlap between the methods was quantified using the Tanimoto index, defined as the ratio of intersecting occupied cells to the union of occupied cells [27, 28]. The resulting normalized overlap matrix provides a quantitative measure of shared chemical space coverage. These regions were visualized using a three-set Venn diagram, with each region annotated by both absolute cell counts and relative percentages.

All analyses were performed using open-source Python libraries, within a single reproducible computational environment (code available at https://github.com/EdgL2/CRUSH). Together, this integrated methodology enabled a multi-level comparison of fragmentation methods, spanning molecular similarity, rule usage, and quantitative chemical space coverage.

### Physicochemical properties of fragments

A CSV file containing fragments and their corresponding fragmentation algorithms (CRUSH, RECAP, or BRICS) was uploaded and read into a pandas DataFrame. Molecular structures were then converted from SMILES strings into molecular objects for further analysis. For each fragment, three physicochemical descriptors were calculated: molecular weight (MW), the number of non-hydrogen atoms (NonHAtoms), the number of sp^3^-hybridized carbon atoms (C_sp3), the number of hydrogen bond donors (HBD), the number of hydrogen bond acceptors (HBA), ClogP, the number of rotatable bonds (NRot), and the polar surface area (PSA) [29], of which dummy atoms were not considered in the calculation. To minimize the influence of extreme values, outliers were removed for each fragmentation group and each descriptor using the interquartile range (IQR) method. This filtering step ensured a fair comparison between the groups while preserving the distribution of the data. The resulting descriptor distributions were visualized using violin plots (see Supplementary material section). Mean and median values were indicated with black internal lines to facilitate visual comparison across groups.

## Results

To evaluate the impact of different fragmentation strategies on fragment generation and chemical space analysis, we performed a comparative analysis across five chemically diverse datasets, namely ChemDiv, LANaPDB, anti-MRSA, Macrocycle-DB, and FooDB. The performance of the three fragmentation algorithms—CRUSH, BRICS, and RECAP—was assessed in terms of the fragment yield, fragment pairing, and the chemical space coverage. Specifically, we compared the fraction of fragments produced by each algorithm, as well as the distribution of paired fragments generated from their parent compounds, using ECFP4 fingerprints (and MACCS keys, see Supplementary material section) and Tanimoto similarity to quantify structural relationships. In addition, we analyzed the number of compounds successfully fragmented by each method and visualized the resulting fragment chemical spaces using UMAP projections combined with KDE. Finally, we quantified the extent of chemical space coverage achieved by the generated fragments, providing an integrated view of their complementarity and overlap across datasets.

**Fig. 1:**
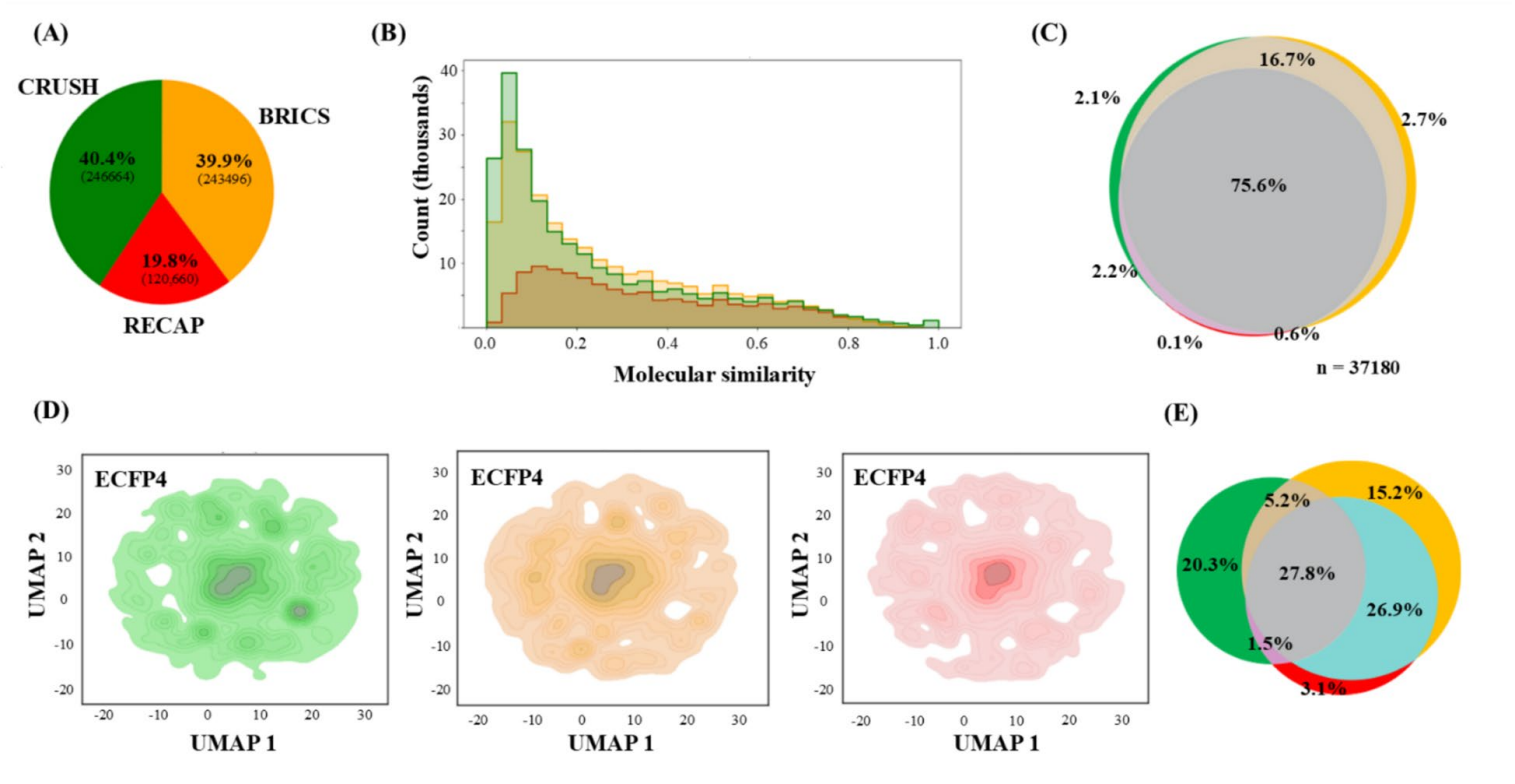
Comparison of fragments generated from ChemDiv. **A** Percentage of fragments generated using different fragmentation algorithms (CRUSH: green; BRICS: orange; RECAP: red); **B** Compound-fragment similarity. Each generated fragment was paired with its respective origin molecule. ECFP4 and the Tanimoto coefficient were used to establish each similarity calculation; **C** Compounds fragmented using different fragmentation algorithms; **D** Chemical spaces generated using different fragmentation algorithms. The maps were generated using kernel density functions from UMAP coordinates; **E** Coverage of chemical spaces of fragments generated using different fragmentation algorithms

Figure 1 summarizes the results obtained for the study of the ChemDiv database. From 41,068 compounds of the ChemDiv database considered for fragmentation, only 3,888 compounds (9.47%) remained uncleaved. If applied individually, CRUSH would fragment the largest number of ChemDiv compounds among all three methods (37,180 out of 41,068; 90.53%). Looking at the compounds fragmented by the three approaches combined, the CRUSH fragmentation algorithm generated approximately twice as many fragments as RECAP and a slightly higher number than BRICS (Fig. 1A). In addition, both CRUSH- and BRICS-derived fragments exhibited lower structural similarity to their parent molecules compared with those generated by RECAP, as assessed using ECFP4 fingerprints and Tanimoto coefficients (Fig. 1B). In this context, 75.6% of all the fragmented molecules in the ChemDiv dataset were decomposed by all three methods, whereas 16.7% were fragmented by both CRUSH and BRICS. CRUSH was found to exclusively fragment 2.1% of molecules compared to both RECAP and BRICS. Finally, the analysis of chemical space coverage of fragments (Fig. 1D and E) revealed that 40.7% of the explored fragment space was accessed exclusively by CRUSH and BRICS, with CRUSH contributing the largest fraction of exclusively covered fragment chemical space (20.3%) among the evaluated fragmentation methods.

**Fig. 2:**
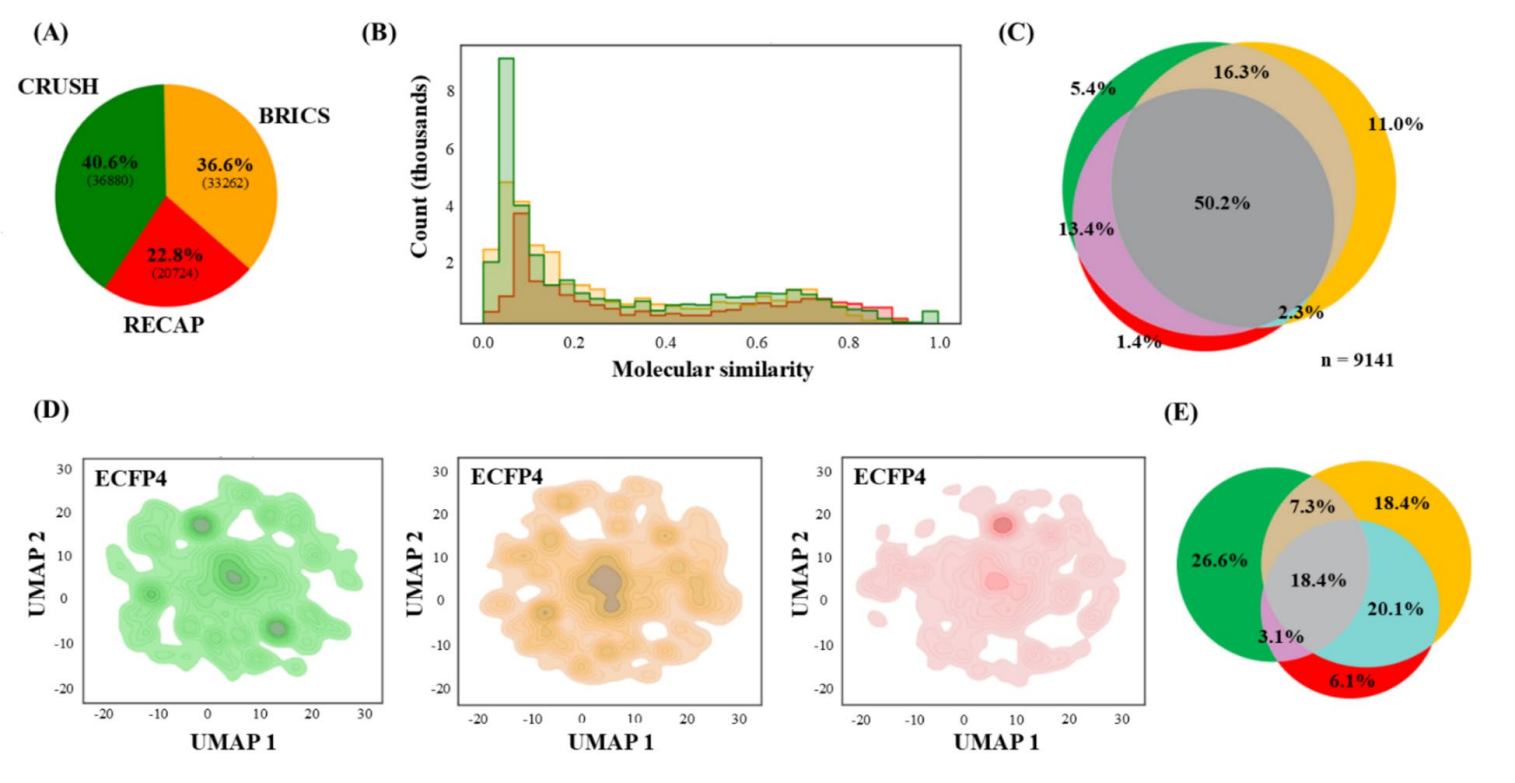
Comparison of fragments generated from LANaPDB v.2. **A** Percentage of fragments generated using different fragmentation algorithms (CRUSH: green; BRICS: orange; RECAP: red); **B** Compound-fragment similarity. Each generated fragment was paired with its respective origin molecule. ECFP4 and the Tanimoto coefficient were used to establish each similarity calculation; **C** Compounds fragmented using different fragmentation algorithms; **D** Chemical spaces generated using different fragmentation algorithms. The maps were generated using kernel density functions from UMAP coordinates; **E** Coverage of chemical spaces of fragments generated using different fragmentation algorithms

Figure 2 summarizes the results obtained for the study of the LANaPDB database. From 13,578 compounds of the LANaPDB v.2 database considered for fragmentation, 4,437 compounds (32.68%) remained uncleaved. If applied individually, CRUSH would fragment the largest number of LANaPDB compounds among all three methods (7,801 out of 13,578; 57.45%). Looking at the compounds fragmented by the three approaches combined, the CRUSH fragmentation algorithm generated approximately twice as many fragments as RECAP and a slightly higher number than BRICS (Fig. 2A). In parallel to this, both CRUSH- and BRICS-derived fragments exhibited lower structural similarity to their parent molecules compared with those generated by RECAP, as assessed using ECFP4 fingerprints and Tanimoto coefficients (Fig. 2B). In this context, 16.3% of the molecules in the LANaPDB dataset were exclusively fragmented by both CRUSH and BRICS, whereas CRUSH exclusively fragmented an additional 5.4% and BRICS 11.0% (Fig. 2C). Finally, analysis of chemical space coverage (Fig. 2D and E) revealed that 52.3% of the explored fragment space was accessed by CRUSH and BRICS combined, with CRUSH and BRICS contributing with 26.6% and 18.4% of the exclusively covered fragment chemical space, respectively.

**Fig. 3:**
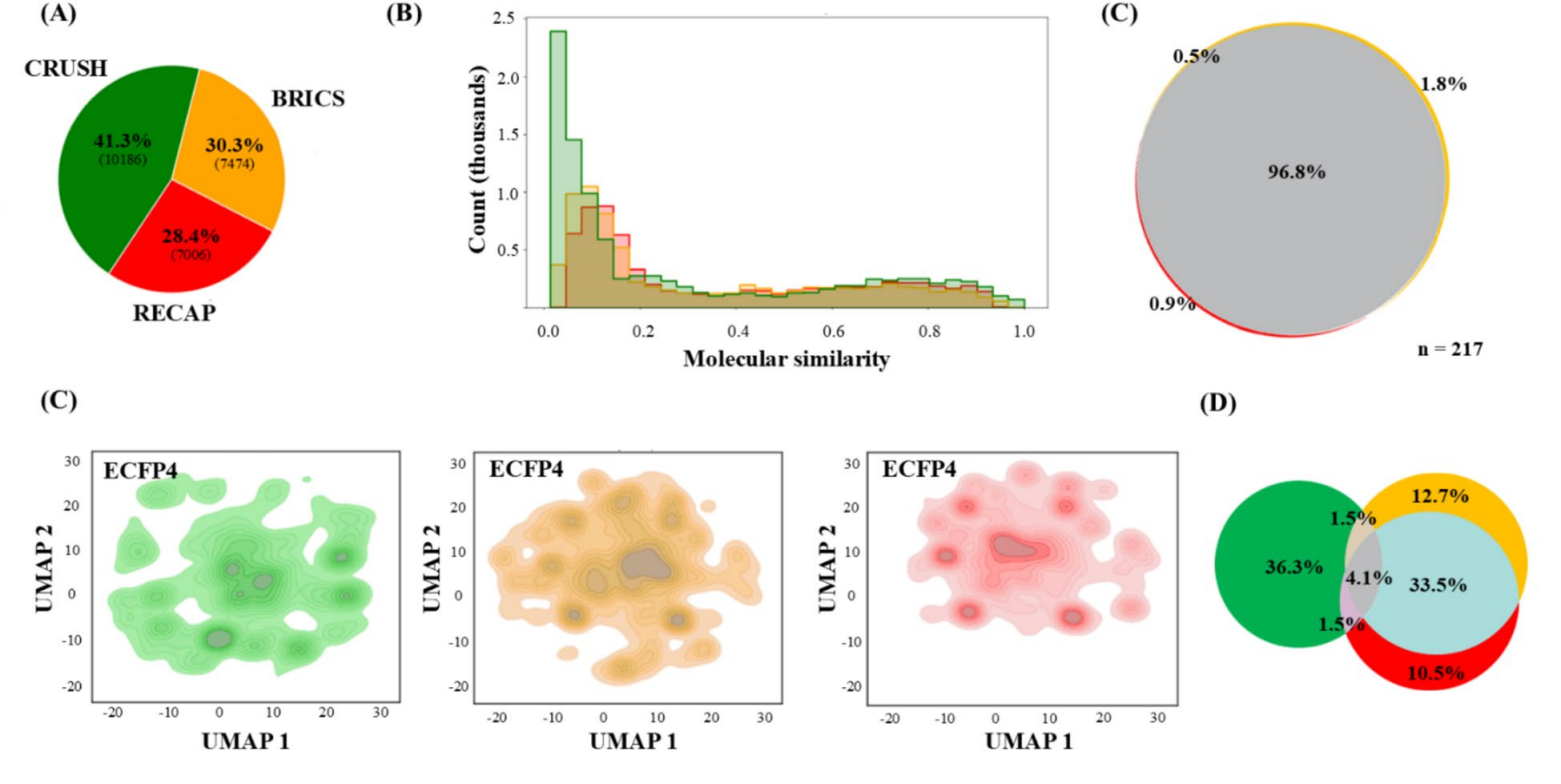
Comparison of fragments generated from anti-MRSA peptides dataset. **A** Percentage of fragments generated using different fragmentation algorithms (CRUSH: green; BRICS: orange; RECAP: red); **B** Compound-fragment similarity. Each generated fragment was paired with its respective origin molecule. ECFP4 and the Tanimoto coefficient were used to establish each similarity calculation; **C** Compounds fragmented using different fragmentation algorithms; **D** Chemical spaces generated using different fragmentation algorithms. The maps were generated using kernel density functions from UMAP coordinates; **E** Coverage of chemical spaces of fragments generated using different fragmentation algorithms

Figure 3 summarizes the results obtained for the study of the anti-MRSA peptides dataset. From 223 compounds of the anti-MRSA peptides dataset database considered for fragmentation, only 6 compounds (2.69%) remained uncleaved. If applied individually, BRICS would fragment the largest number of anti-MRSA peptides among all three methods (215 out of 223; 96.41%). Looking at the compounds fragmented by the three approaches combined, the CRUSH fragmentation algorithm generated a higher number of fragments compared with RECAP and BRICS (Fig. 3A). In parallel to this, CRUSH-derived fragments exhibited lower structural similarity to their parent molecules compared with those generated by RECAP or BRICS, as assessed using ECFP4 fingerprints and Tanimoto coefficients (Fig. 3B). Notably, we observed that 96.8% of compounds were fragmented by all three fragmentation algorithms, whereas CRUSH cleaved only 0.5% of exclusive compounds (Fig. 3C). Finally, analysis of fragment chemical space coverage revealed that 50.5% of the explored fragment space was accessed exclusively by CRUSH and BRICS, with CRUSH contributing 36.3% of the uniquely covered chemical space among the evaluated fragmentation methods (Fig. 3D and E). Although most of the molecules were fragmented by all three methods, the quantity and diversity of fragments generated by CRUSH are superior to RECAP and BRICS.

**Fig. 4:**
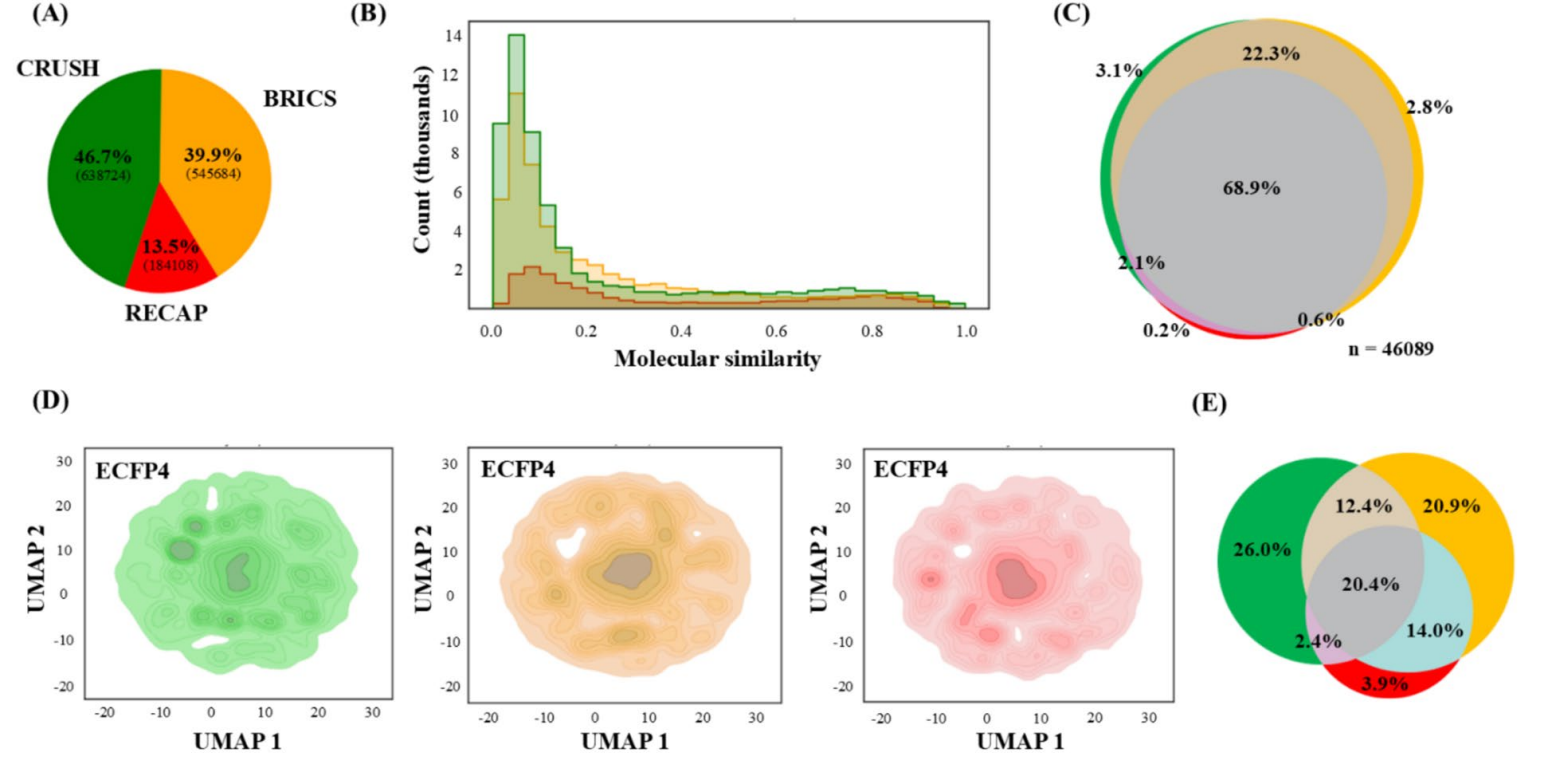
Comparison of fragments generated from Macrocycle-DB. **A** Percentage of fragments generated using different fragmentation algorithms (CRUSH: green; BRICS: orange; RECAP: red); **B** Compound-fragment similarity. Each generated fragment was paired with its respective origin molecule. ECFP4 and the Tanimoto coefficient were used to establish each similarity calculation; **C** Compounds fragmented using different fragmentation algorithms; **D** Chemical spaces generated using different fragmentation algorithms. The maps were generated using kernel density functions from UMAP coordinates; **E** Coverage of chemical spaces of fragments generated using different fragmentation algorithms

Figure 4 summarizes the results obtained for the study of the Macrocycle-DB. From 46,089 compounds of the Macrocycle-DB peptides database considered for fragmentation, no compounds remained uncleaved. If applied individually, CRUSH would fragment the largest number of Macrocycle-DB compounds among all three methods (44,428 out of 46,089; 96.39%). Looking at the compounds fragmented by the three approaches combined, the CRUSH fragmentation algorithm generated approximately three times more fragments than RECAP and a slightly greater proportion than BRICS (Fig. 4A). In parallel to this, both CRUSH- and BRICS-derived fragments exhibited lower structural similarity to their parent molecules compared with those generated by RECAP, as assessed using ECFP4 fingerprints and Tanimoto coefficients (Fig. 4B). In this context, 22.3% of the molecules in the Macrocycle-DB were fragmented by both CRUSH and BRICS, whereas CRUSH exclusively contributed with fragmenting 3.1% of the dataset (Fig. 4C). Finally, analysis of fragment chemical space coverage revealed that 59.3% of the explored space was accessed exclusively by CRUSH and BRICS, with CRUSH contributing the largest fraction of uniquely covered fragment chemical space (26.0%) among the evaluated fragmentation methods (Fig. 4D and E).

**Fig. 5:**
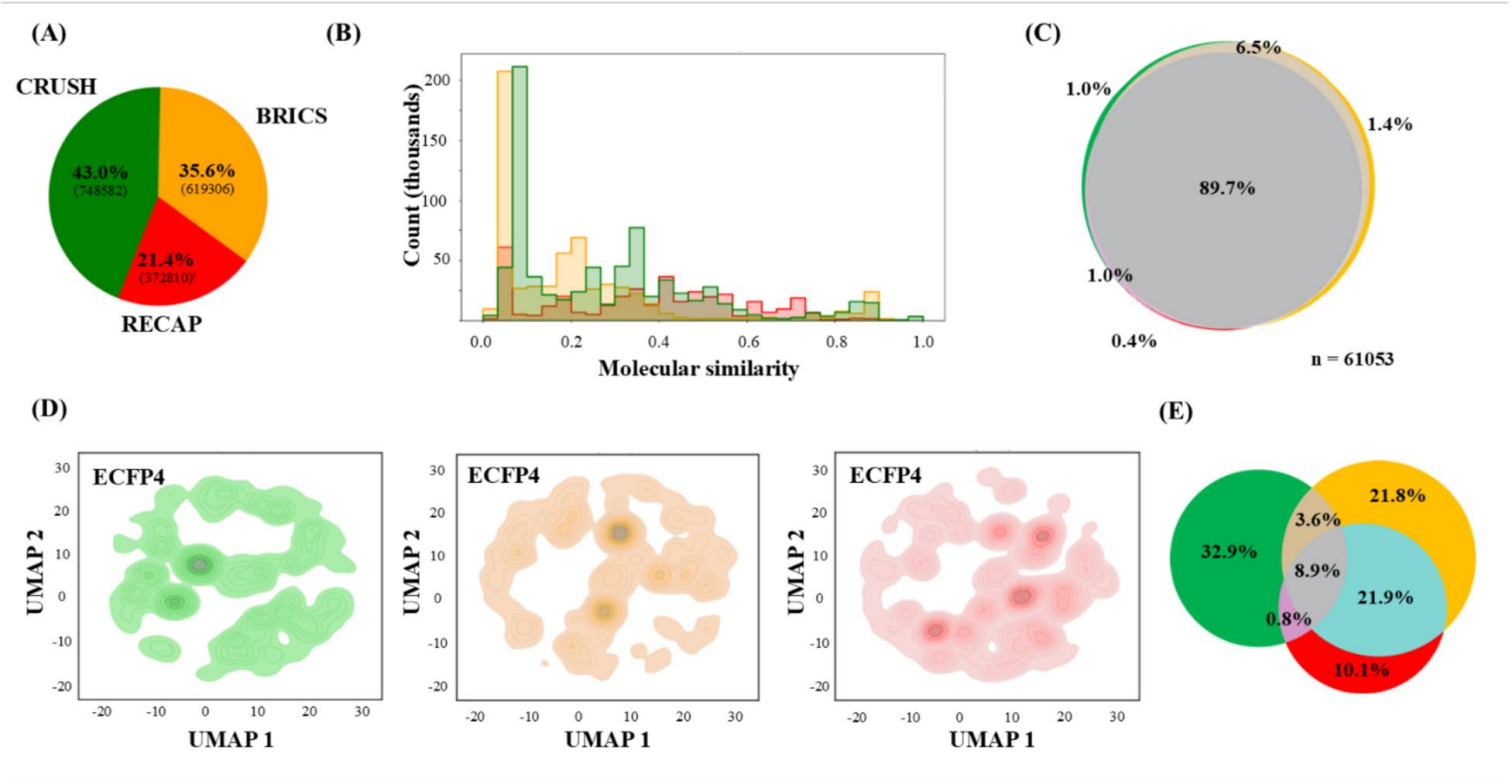
Comparison of fragments generated from FooDB. **A** Percentage of fragments generated using different fragmentation algorithms (CRUSH: green; BRICS: orange; RECAP: red); **B** Compound-fragment similarity. Each generated fragment was paired with its respective origin molecule. ECFP4 and the Tanimoto coefficient were used to establish each similarity calculation; **C** Compounds fragmented using different fragmentation algorithms; **D** Chemical spaces generated using different fragmentation algorithms. The maps were generated using kernel density functions from UMAP coordinates; **E** Coverage of chemical spaces of fragments generated using different fragmentation algorithms

Figure 5 summarizes the results obtained for the study of the FooDB database. From 70,477 compounds of the FooDB database considered for fragmentation, only 9,424 compounds (13.37%) remained uncleaved. If applied individually, CRUSH would fragment the largest number of FooDB compounds among all three methods (59,984 out of 70,477; 85.11%). Looking at the compounds fragmented by the three approaches combined, the CRUSH fragmentation algorithm generated approximately twice as many fragments as RECAP and a higher number than BRICS (Fig. 5A). In parallel to this, both CRUSH- and BRICS-derived fragments exhibited lower structural similarity to their parent molecules compared with those generated by RECAP, as assessed using ECFP4 fingerprints and Tanimoto coefficients (Fig. 5B). In this context, 6.5% of the molecules in FooDB were fragmented by both CRUSH and BRICS, whereas CRUSH exclusively fragmented an additional 1.0% of compounds (Fig. 5C). Finally, the analysis of fragment chemical space coverage revealed that 58.3% of the explored fragment space was accessed exclusively by CRUSH and BRICS, with CRUSH contributing 32.9% of uniquely covered fragment space among the evaluated fragmentation methods (Fig. 5D and E).

The results of the fragment chemical space coverage were similar when produced using low-dimensional MACCS keys (166-bit), yielding comparable outcomes with the high-dimensional ECFP4 fingerprint (Figures S1–S5 in the Supplementary material). Importantly, CRUSH and BRICS generated fragments that explored regions of chemical space not accessed by RECAP. Notably, CRUSH-derived fragments consistently occupied distinct chemical space regions exclusive to this method, regardless of whether the fragmented compounds corresponded to drug-like molecules, natural products, peptides, macrocycles, or food-related compounds. Moreover, the unique contribution of CRUSH was often greater than that of RECAP and BRICS across all of the databases.

Additionally, the fragments generated by each fragmentation method were evaluated based on the descriptors related to their size, relative complexity, and physicochemical properties (Figures S6–S10 in the Supplementary material). The fragment-based properties were analyzed across all five databases to assess their compliance with the Rule of 3 (Ro3: MW ≤ 300 Da, HBD ≤ 3, HBA ≤ 3, ClogP ≤ 3, NRot ≤ 3, PSA ≤ 60 Å^2^), a widely used criterion to define fragment-like chemical space. In general, CRUSH and BRICS produced fragments with lower values of HBD, HBA, ClogP, NRot, and PSA compared to RECAP (Figures S6–S10). Accordingly, fragments generated by CRUSH and BRICS showed property distributions more consistently within Ro3 thresholds, particularly across the ChemDiv, FooDB, and LANaPDB2 databases, where median values for all evaluated properties generally satisfied the Ro3 criteria [30, 31]. In contrast, RECAP fragments exhibited broader distributions with notably higher MW, NRot, and ClogP values, indicating lower overall Ro3 compliance. This difference was especially pronounced in FooDB, where RECAP fragments presented a median NRot of 13 and a median ClogP of 5, both substantially exceeding the Ro3 limits. For databases containing structurally complex parent compounds, such as Macrocycles-DB and the anti-MRSA dataset, all three fragmentation methods produced subsets of fragments violating multiple Ro3 criteria. Such result reflects the intrinsic complexity of the source molecules rather than methodological limitation. Taken together, these results suggest that CRUSH and BRICS are better suited for generating fragment-like building blocks conforming to the Ro3, while RECAP tends to retain larger and more complex structural features from the parent compounds.

## Discussion

A meaningful comparison of molecular fragmentation algorithms requires the use of consistent metrics that capture not only the quantity of fragments generated, but also their diversity, structural characteristics, and spatial distribution within chemical space [32, 33]. In this work, fragment count, chemical space coverage, and fragment-level descriptors were jointly employed to enable an unbiased assessment of CRUSH, BRICS, and RECAP across chemically diverse datasets. The concordant trends observed when using fingerprints of different resolutions (MACCS keys and ECFP4) reinforce the robustness of these metrics and indicate that the reported differences are intrinsic to the fragmentation strategies rather than artifacts of a specific molecular representation [34].

The observed distribution of fragments across chemical space suggests that CRUSH and BRICS are better interpreted as complementary rather than strictly competing approaches. Across all evaluated datasets, both methods consistently accessed regions of chemical space not covered by RECAP, with a substantial fraction of fragment space jointly populated by CRUSH and BRICS, alongside method-specific exclusive regions. Notably, while CRUSH contributed the largest proportion of uniquely covered chemical space, BRICS systematically populated overlapping yet chemically meaningful regions, reflecting its retrosynthetically guided design. This partial overlap, combined with distinct exclusive contributions, indicates that each method captures different aspects of molecular structure and decomposition. Consequently, integrating fragments derived from both CRUSH and BRICS could further enhance chemical space coverage, balancing the exploration of novel chemotypes (driven by CRUSH) with chemically intuitive and synthetically grounded substructures (provided by BRICS). Such a combined strategy may therefore offer added value in applications requiring both diversity and chemical interpretability.

Fragment count alone provides limited insight into the practical utility of a fragmentation method, as a larger number of fragments does not necessarily translate into increased chemical diversity or exploratory power [5]. By integrating chemical space coverage analyses, we demonstrate that CRUSH and BRICS outperform RECAP in accessing underexplored regions of chemical space. Notably, CRUSH-derived fragments consistently occupy exclusive regions, regardless of the chemical origin of the parent compounds, including drug-like molecules, natural products, peptides, macrocycles, and food-related compounds. This finding highlights the importance of spatial metrics in chemoinformatics, such as grid-based occupancy and overlap indices, to reveal qualitative differences that are not apparent from fragment abundance alone. A potential confounding factor in the interpretation of similarity and chemical space coverage metrics is the underlying fragment size distribution generated by each method. In particular, smaller fragments, such as those predominantly produced by CRUSH and BRICS, may inherently exhibit lower structural similarity to their parent compounds and occupy broader regions of chemical space due to reduced structural constraints. This effect could lead to a method-dependent inflation of diversity and coverage metrics, thereby partially biasing comparisons against methods like RECAP, which tend to generate larger and more complex fragments. Therefore, an exhaustive assessment incorporating size-controlled analyses—such as stratification by fragment size or normalization of similarity scores—represents an important direction for our future work.

The observed differences in fragment size and complexity further emphasize the downstream implications of fragmentation choice [30, 31]. CRUSH and BRICS generate smaller and less complex fragments, characterized by lower values across all evaluated physicochemical parameters compared with RECAP. Such properties are particularly advantageous in fragment-based drug discovery, where smaller, simpler fragments are more amenable to efficient sampling, binding mode optimization, and synthetic elaboration [35–38]. In contrast, the larger and more complex fragments produced by RECAP may be better suited for applications focused on retrosynthetic analysis or scaffold dissection of drug-like compounds using established medicinal chemistry synthesis rules [39], but may limit chemical space exploration within a wider organic chemistry landscape and application in diversity-driven workflows.

On the other hand, BRICS had been designed around retrosynthetically meaningful bond disconnections guided by chemically intuitive rules [40], which results in fragments that are generally smaller and less complex than those produced by RECAP, while still preserving chemically reasonable functional group environments [41, 42]. This intermediate position between CRUSH and RECAP is reflected in both fragment size descriptors and chemical space coverage patterns.

However, despite these advantages, BRICS does not achieve the same level of compound coverage and fragment chemical space exclusivity as CRUSH. The chemical space coverage analysis indicates that, although BRICS expands beyond the regions accessed by RECAP, a substantial fraction of its fragments occupies areas overlapping with other methods. This suggests that BRICS favors the systematic decomposition of molecules into chemically familiar substructures rather than exploiting underrepresented regions of chemical space. As a result, BRICS-derived fragment libraries may be biased toward well-established chemotypes, which can be advantageous for lead optimization but potentially limiting for exploratory or diversity-driven discovery efforts, especially in the study of natural products, canonical and non-canonical peptides, macrocycles, and alimentary compounds.

The dependence of these results on the selected fragmentation scheme should also be considered. In particular, the breadth and granularity of the rule set—such as the explicit inclusion of diverse bond disconnections spanning carbon–heteroatom linkages, aromatic–aliphatic interfaces, charged centers, and multiple functional group environments (*e.g.*, amides, sulfonamides, carbamates, disulfides, and aryl–heteroatom bonds)—directly influence both the number of generated fragments and their distribution in chemical space. In this study, the configuration yielding the largest number of fragments was selected to standardize comparisons and enhance output resolution; however, alternative configurations would likely decrease fragmentation density and, consequently, limit chemical space coverage. Nonetheless, the relative differences observed between CRUSH, BRICS, and RECAP are primarily driven by their underlying fragmentation logic and are therefore expected to be preserved across different configuration sets. For this reason, a prospective systematic evaluation across multiple fragmentation configurations, schemes, and complementary diverse datasets would be valuable to further assess robustness and generality of fragmentation approaches.

**Fig. 6:**
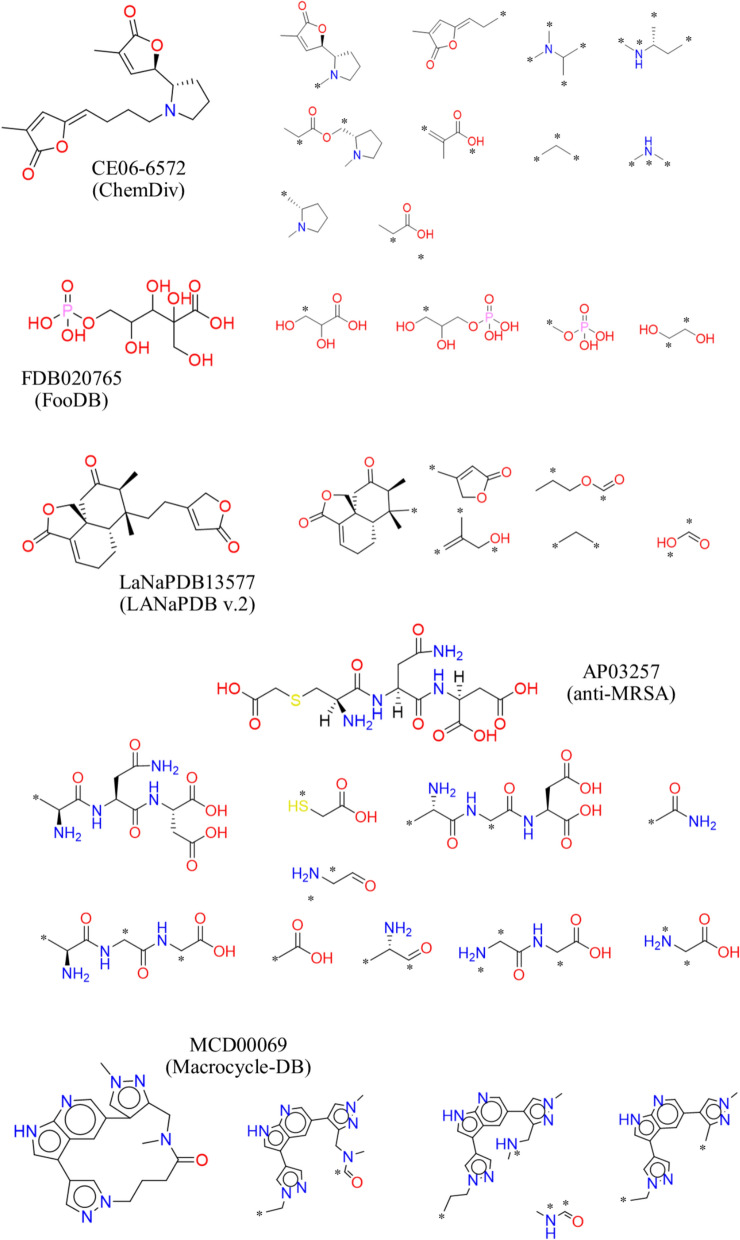
Representative examples of fragments generated with CRUSH in this work. The identifiers of each originating compound are displayed along with its source database, as well as the complete list of its generated complementary fragments. The atoms marked with * in each fragment indicate the sites where one or more fragmentation rules were used

In parallel, CRUSH explicitly incorporates a fine-grained chemical interpretation of bond environments, including aliphatic and aromatic C–C bonds, heteroatom–carbon linkages, exocyclic aromatic substitutions, quaternary ammonium centers, alkynes, disulfides, and sulfonate esters (Table 1). By doing so, CRUSH captures chemically distinct subunits that RECAP frequently overlooks and BRICS coarsely represents. Importantly, CRUSH distinguishes between ring and non-ring contexts, as well as between saturated and unsaturated systems, leading to fragments that better reflect bonding topology (Fig. 6).

Importantly, while CRUSH enables the identification of chemically meaningful substructures and facilitates the exploration of underrepresented regions of chemical space, it does not explicitly incorporate synthesizability constraints in the same manner as retrosynthetically driven approaches such as RECAP. This limitation should be considered when using CRUSH for hypothesis generation, particularly in contexts where synthetic feasibility is a primary requirement. In this regard, CRUSH is best viewed as a complementary strategy that prioritizes structural diversity and chemical novelty over immediate synthetic accessibility. Its strengths are therefore especially evident in data-driven applications, including machine learning workflows, where fragment-based representations can enhance data density, improve interpretability, and ultimately contribute to model performance [6]. Consequently, hypotheses derived from CRUSH are more appropriately applied during early-stage exploratory phases, where the identification of novel chemotypes precedes subsequent filtering or refinement using synthesizability-aware methodologies.

Although CRUSH is not explicitly designed from a retrosynthetic perspective, the fragmentation scheme preserves complementary bond environments that, in principle, enable the reconstruction of the original parent molecule through the recombination of matched fragment pairs generated during the cleavage process. This intrinsic re-constructability arises from the consistent encoding of bond types and local chemical contexts at the fragmentation sites, which maintains structural compatibility between fragments. While this does not guarantee synthetic feasibility in the same sense as rule-based approaches such as BRICS or RECAP, it provides a coherent framework for fragment recombination *in silico*. In this context, CRUSH-derived fragments are particularly well suited for applications involving systematic fragment recombination, enumeration of novel chemotypes, and data augmentation workflows.

Overall, the performance of CRUSH, BRICS, and RECAP in this analysis underscores the importance of aligning the fragmentation strategy with the intended downstream application. For example, for tasks prioritizing maximal chemical space exploration, chemical diversity, and fragment novelty, approaches such as CRUSH may offer complementary advantages. These observations reinforce the need for systematic, metric-driven comparisons when selecting fragmentation methods for specific drug discovery and chemical biology pipelines.

**Fig. 7:**
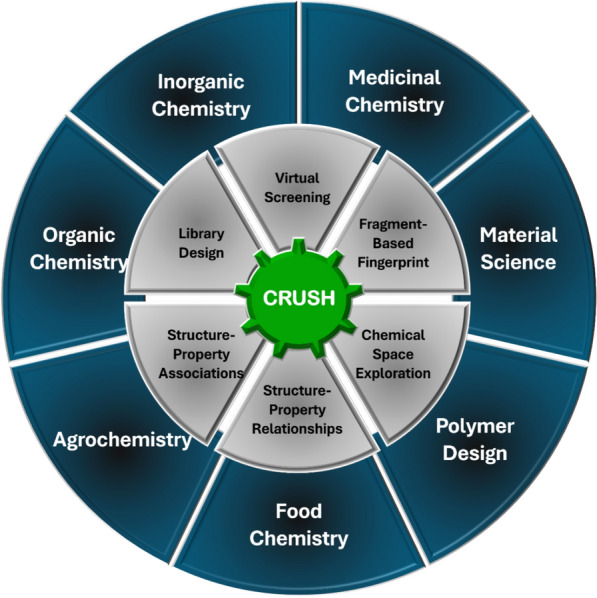
New frontiers of the CRUSH fragmentation method use and future areas for application

Importantly, the consistent tendencies observed across datasets of varying chemical nature suggest that the influence of fragmentation strategy extends beyond dataset-specific biases. Methods like CRUSH, which prioritize broad and unique chemical space coverage, are therefore well positioned for applications involving *de novo* design, virtual screening, generation of chemically diverse fragment libraries, and the development of fragment-based molecular fingerprints, within a wide range of chemical industries (Fig. 7).

## Conclusions

This study introduces CRUSH as a chemically representative and application-oriented molecular fragmentation method, which demonstrates higher performance through a comprehensive comparison with the established BRICS and RECAP approaches. CRUSH was conceived to overcome key limitations of traditional fragmentation strategies by adopting a fine-grained, chemistry-aware bond disconnection philosophy that extends beyond classical retrosynthetic rules, enabling the systematic deconvolution of complex molecular structures into chemically meaningful subunits.

Through a unified evaluation framework applied to five chemically diverse datasets, we show that the introduction of CRUSH results in a consistent increase in fragment yield and, more importantly, in a markedly broader and more exclusive exploration of chemical space. Across all datasets (based on drug-like, natural products, peptides, macrocycles, and alimentary compounds), CRUSH-derived fragments accessed regions of chemical space not reached by BRICS or RECAP, a behavior that was preserved when using both low- and high-resolution molecular fingerprints (MACCS keys and ECFP4, respectively). These findings indicate that the advantages of CRUSH are intrinsic to its fragmentation logic and not dependent on a particular molecular representation or dataset composition.

Structurally, CRUSH generates fragments that are smaller and less complex (as measured by sp^3^ carbon content) than those produced by RECAP and, in many cases, comparable to or simpler than those obtained with BRICS. This structural profile is particularly favorable for fragment-based and diversity-driven workflows, where reduced molecular complexity facilitates efficient chemical space sampling, fragment recombination, and downstream optimization. In contrast to BRICS, which remains partially constrained by retrosynthetic reconnection rules, CRUSH prioritizes the explicit capture of chemically distinct substructures, leading to higher fragment novelty and chemical space exclusivity.

Together, these results establish CRUSH as a robust and versatile fragmentation framework that complements and, in exploratory contexts, surpasses existing methods. By enabling fine-resolution chemical space deconvolution and generating chemically interpretable, diverse fragment libraries, CRUSH provides a strong foundation for applications in fragment-based discovery, virtual screening, *de novo* design, and the development of fragment-based molecular representations across multiple domains of chemistry, as schematically summarized in Fig. 7.

## Supplementary Information

Supplementary material 1.

## Data Availability

All data supporting this research are available on the Zenodo platform (10.5281/zenodo.18665641). The code for molecular fragmentation using the described cleavage rules, along with scripts for result analysis, is available on GitHub (https://github.com/EdgL2/CRUSH).
