# Supplementary material for: CRUSH—Cleavage Rules Using SMIRKS Heuristics: an enhanced molecular fragmentation algorithm
Source: J Cheminform. 2026 Jun 13;18:78. doi: 10.1186/s13321-026-01239-w (PMC13267520; doi:10.1186/s13321-026-01239-w)
Supplement: Supplementary file 1 — Supplementary material 1. [file 13321_2026_1239_MOESM1_ESM.docx]

**Supplementary Material**

**CRUSH - Cleavage Rules Using SMIRKS Heuristics: An Enhanced Molecular Fragmentation Algorithm**

Edgar López-López^1,2^, José L. Medina-Franco^1^, Filip Miljković^2,3*^

^1^DIFACQUIM Research Group, Department of Pharmacy, School of Chemistry, Universidad Nacional Autónoma de México, Avenida Universidad 3000, Mexico City 04510, Mexico

^2^Department of Pharmaceutical Biosciences, Uppsala University, Box 591, 75124, Uppsala, Sweden

^3^Biopharma Chemistry, Discovery Sciences, Biopharmaceuticals R&D, AstraZeneca, Pepparedsleden 1, 43183, Mölndal, Sweden

*Corresponding author: Phone: +46-73-699-3320, E-mail: filip.miljkovic@uu.se; filip.miljkovic@astrazeneca.com, ORCID: 0000-0001-5365-505X.

**Index**

| **Content** | **Page** |
| --- | --- |
| Figure S1. Comparison of fragments generated from ChemDiv | S3 |
| Figure S2. Comparison of fragments generated from LANaPDB v.2 | S3 |
| Figure S3. Comparison of fragments generated from anti-MRSA peptides dataset | S4 |
| Figure S4. Comparison of fragments generated from Macrocycle-DB | S4 |
| Figure S5. Comparison of fragments generated from FooDB | S5 |
| Figure S6. Comparison of fragments' properties generated from ChemDiv | S5 |
| Figure S7. Comparison of fragments' properties generated from LANaPDB v.2 | S6 |
| Figure S8. Comparison of fragments' properties generated from anti-MRSA peptides dataset | S7 |
| Figure S9. Comparison of fragments' properties generated from Macrocycle-DB | S8 |
| Figure S10. Comparison of fragments' properties generated from FooDB | S9 |


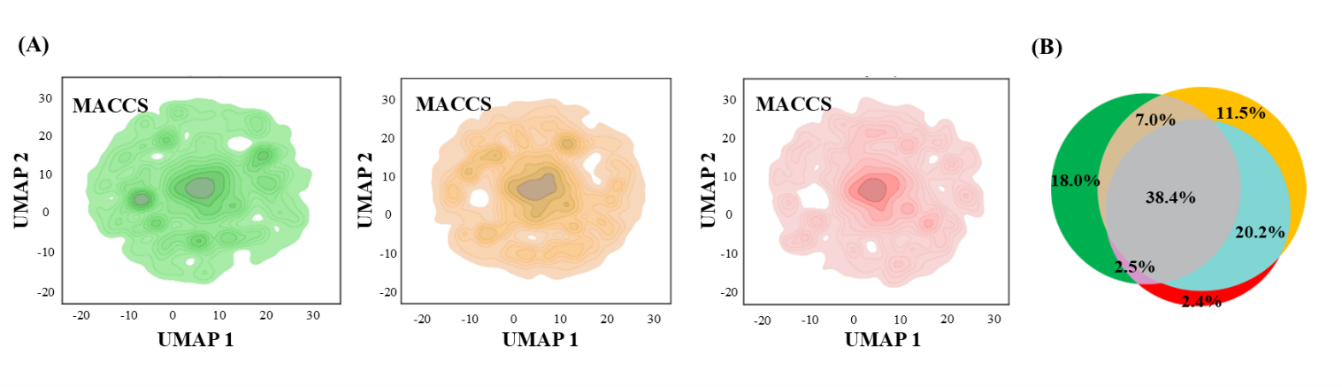


**Figure S1.** Comparison of fragments generated from ChemDiv. (**A**) Chemical spaces generated using different fragmentation algorithms. Each generated fragment was paired with its respective origin molecule. MACCS keys are used as molecular fingerprints. The maps were generated using kernel density functions from UMAP coordinates; (**B**) Coverage of chemical spaces of fragments generated using different fragmentation algorithms.


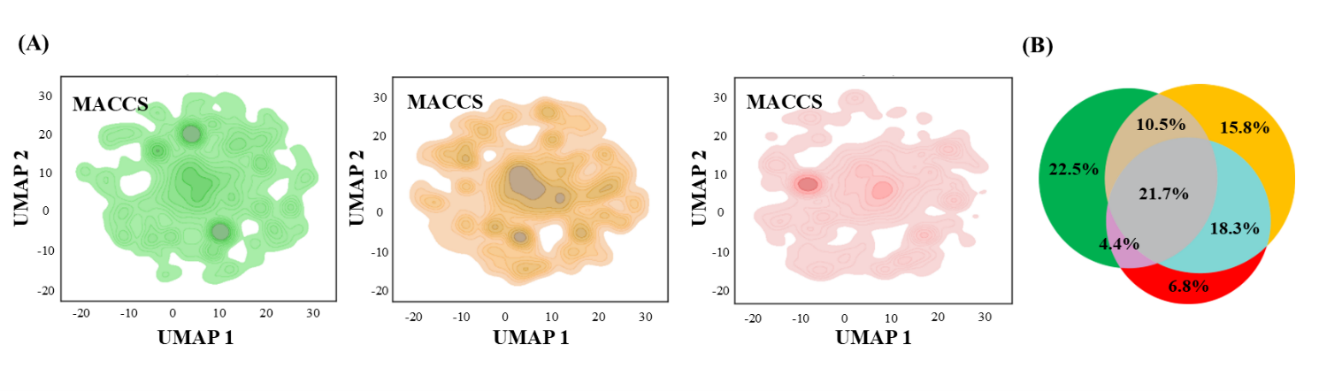


**Figure S2.** Comparison of fragments generated from LANaPDB v.2. (**A**) Chemical spaces generated using different fragmentation algorithms. Each generated fragment was paired with its respective origin molecule. MACCS keys are used as molecular fingerprints. The maps were generated using kernel density functions from UMAP coordinates; (**B**) Coverage of chemical spaces of fragments generated using different fragmentation algorithms.


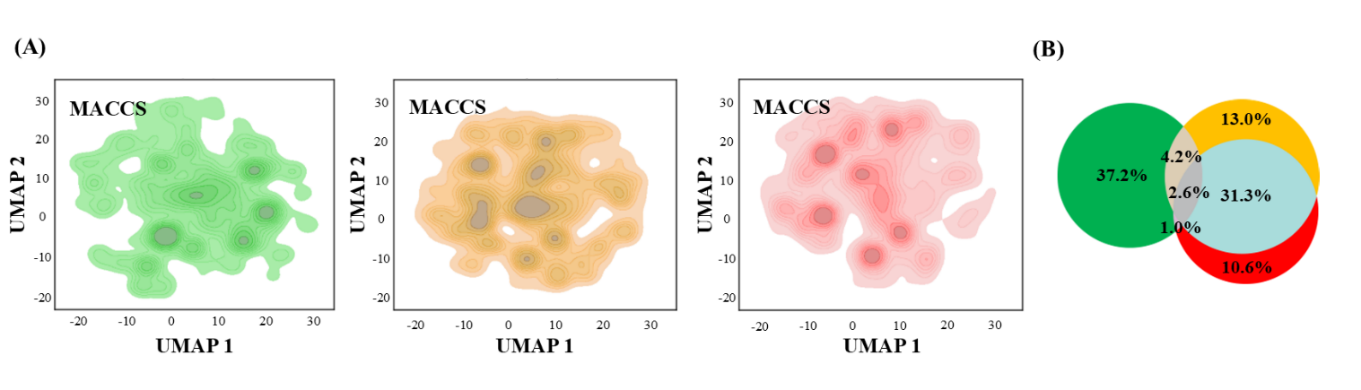


**Figure S3.** Comparison of fragments generated from anti-MRSA peptides dataset. (**A**) Chemical spaces generated using different fragmentation algorithms. Each generated fragment was paired with its respective origin molecule. MACCS keys are used as molecular fingerprints. The maps were generated using kernel density functions from UMAP coordinates; (**B**) Coverage of chemical spaces of fragments generated using different fragmentation algorithms.


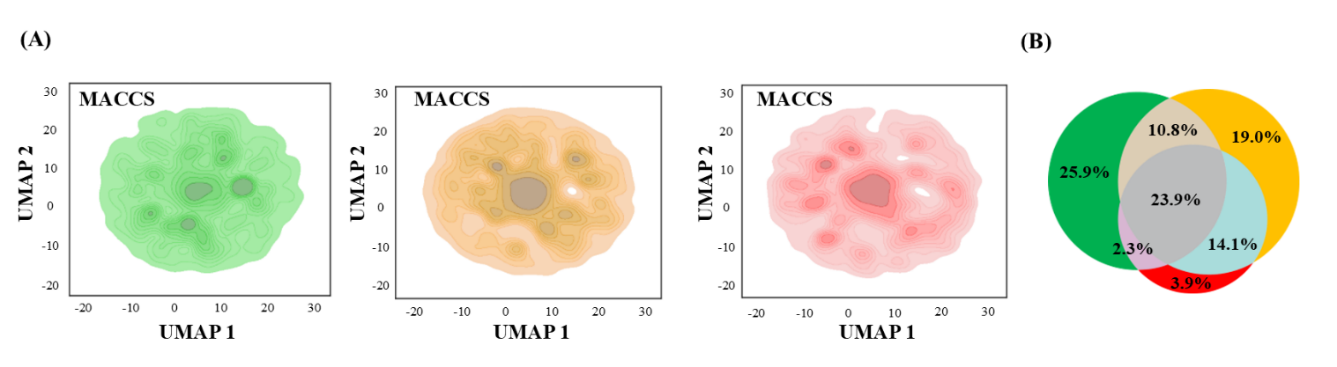


**Figure S4.** Comparison of fragments generated from Macrocycle-DB. (**A**) Chemical spaces generated using different fragmentation algorithms. Each generated fragment was paired with its respective origin molecule. MACCS keys are used as molecular fingerprints. The maps were generated using kernel density functions from UMAP coordinates; (**B**) Coverage of chemical spaces of fragments generated using different fragmentation algorithms.


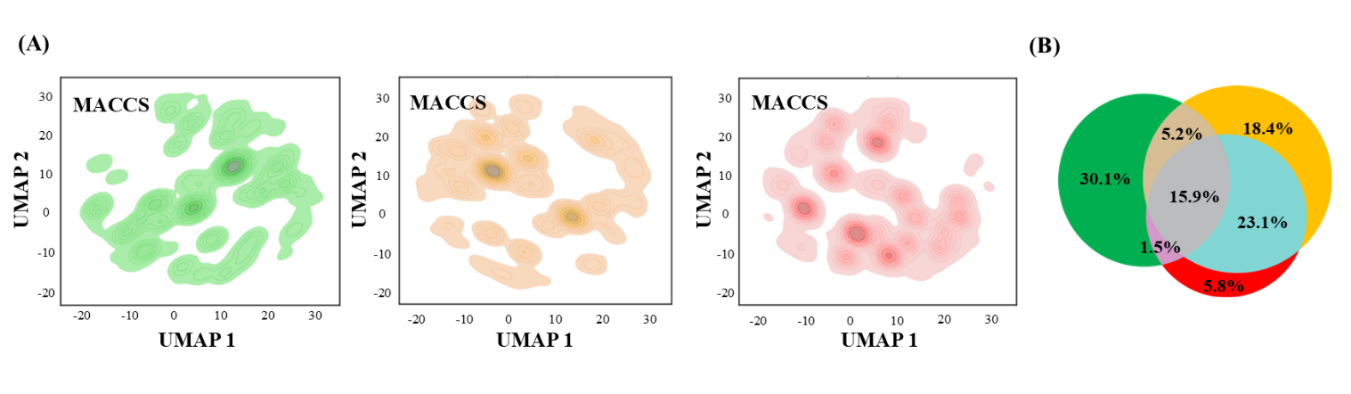


**Figure S5.** Comparison of fragments generated from FooDB. (**A**) Chemical spaces generated using different fragmentation algorithms. Each generated fragment was paired with its respective origin molecule. MACCS keys are used as molecular fingerprints. The maps were generated using kernel density functions from UMAP coordinates; (**B**) Coverage of chemical spaces of fragments generated using different fragmentation algorithms.


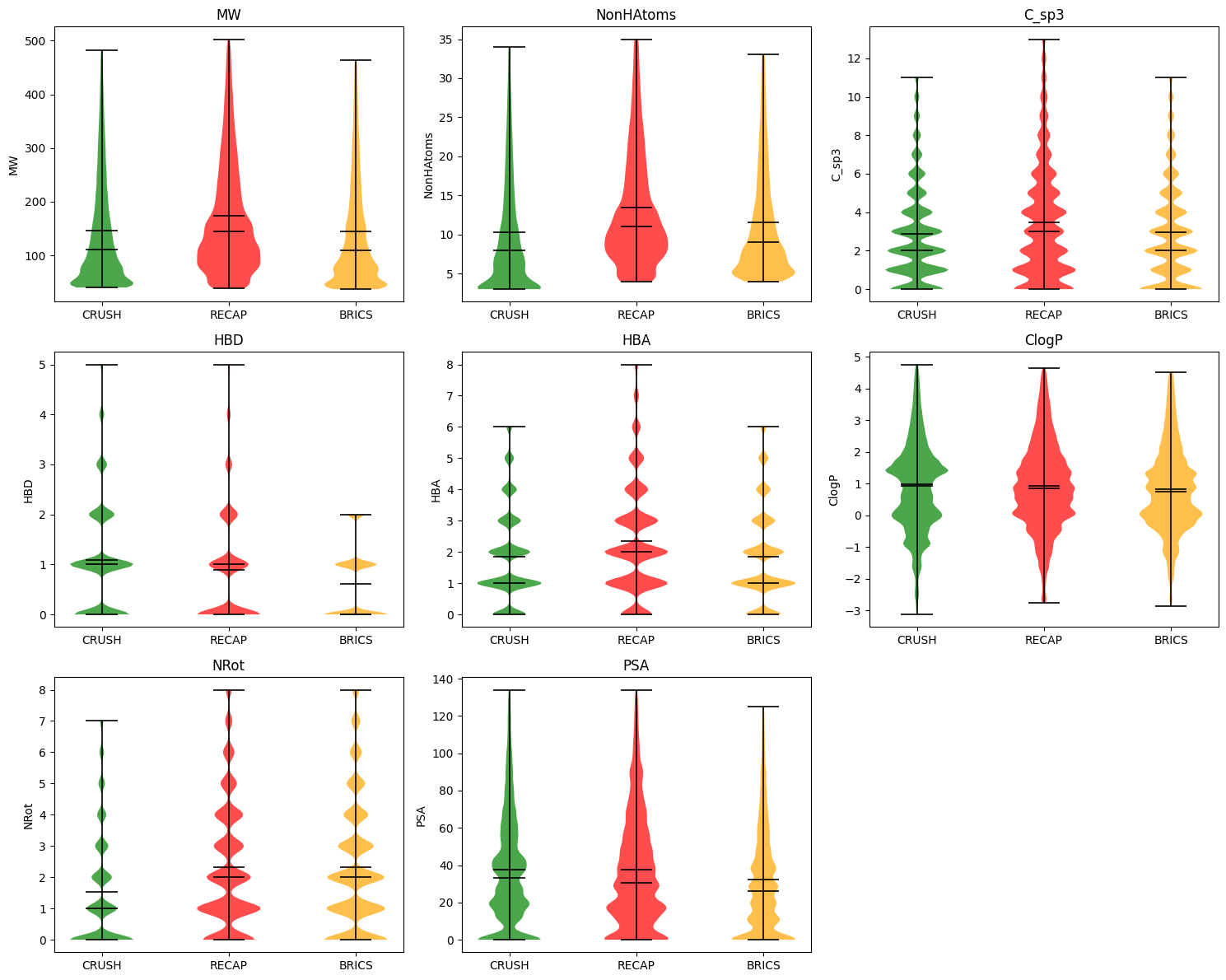


**Figure S6.** Comparison of fragments' properties generated from ChemDiv.


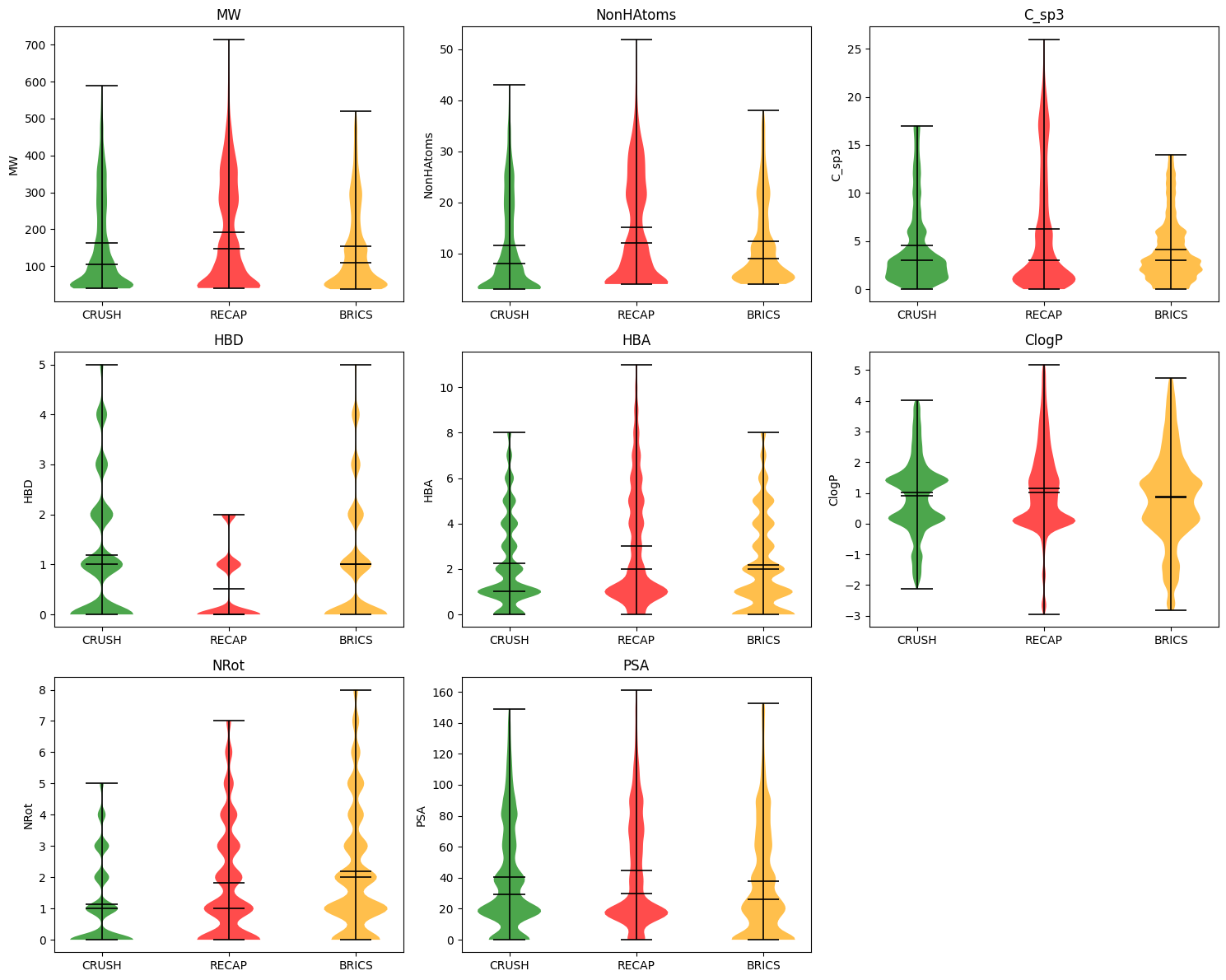


**Figure S7.** Comparison of fragments' properties generated from LANaPDB v.2.

**
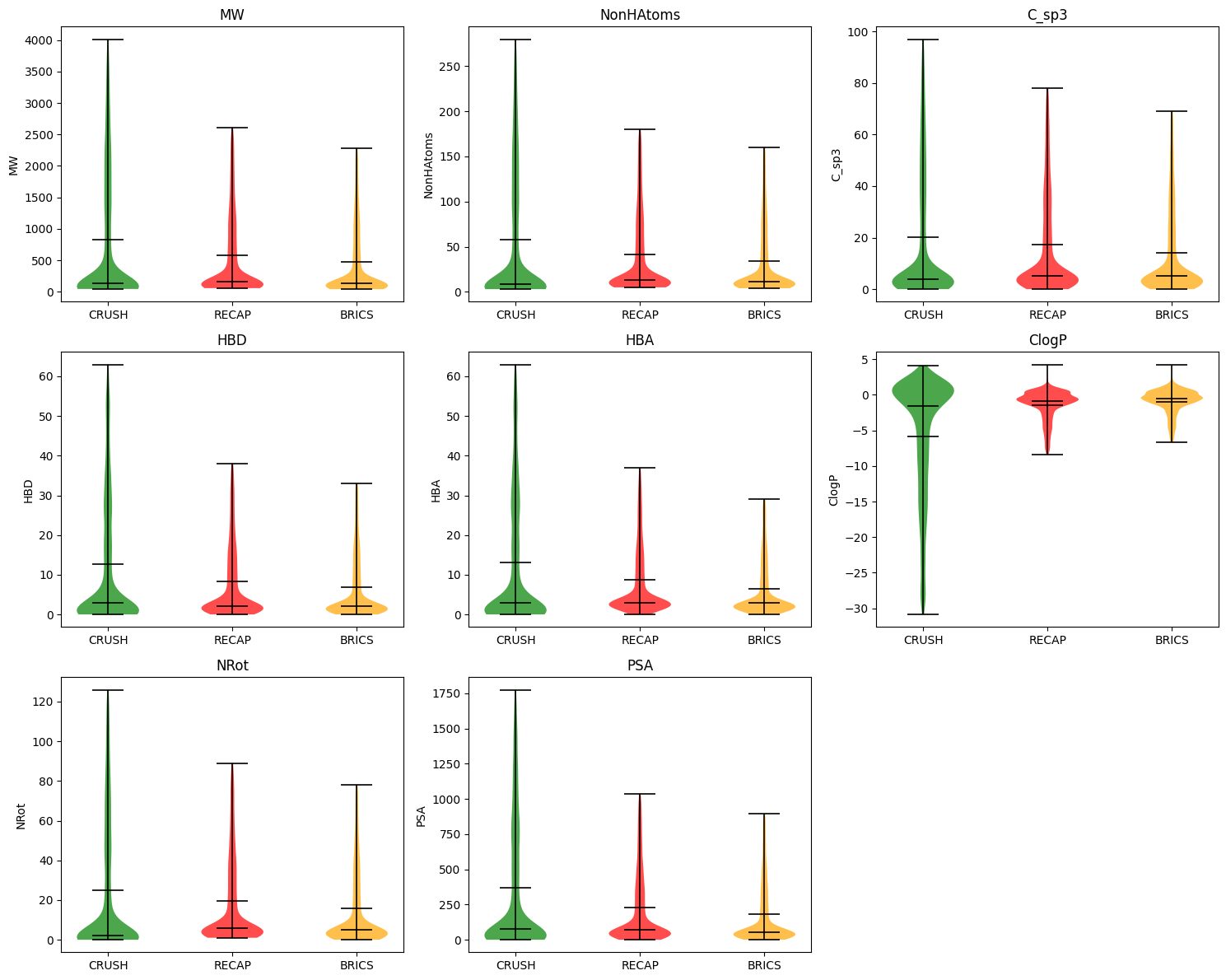
**

**Figure S8.** Comparison of fragments' properties generated from anti-MRSA dataset.


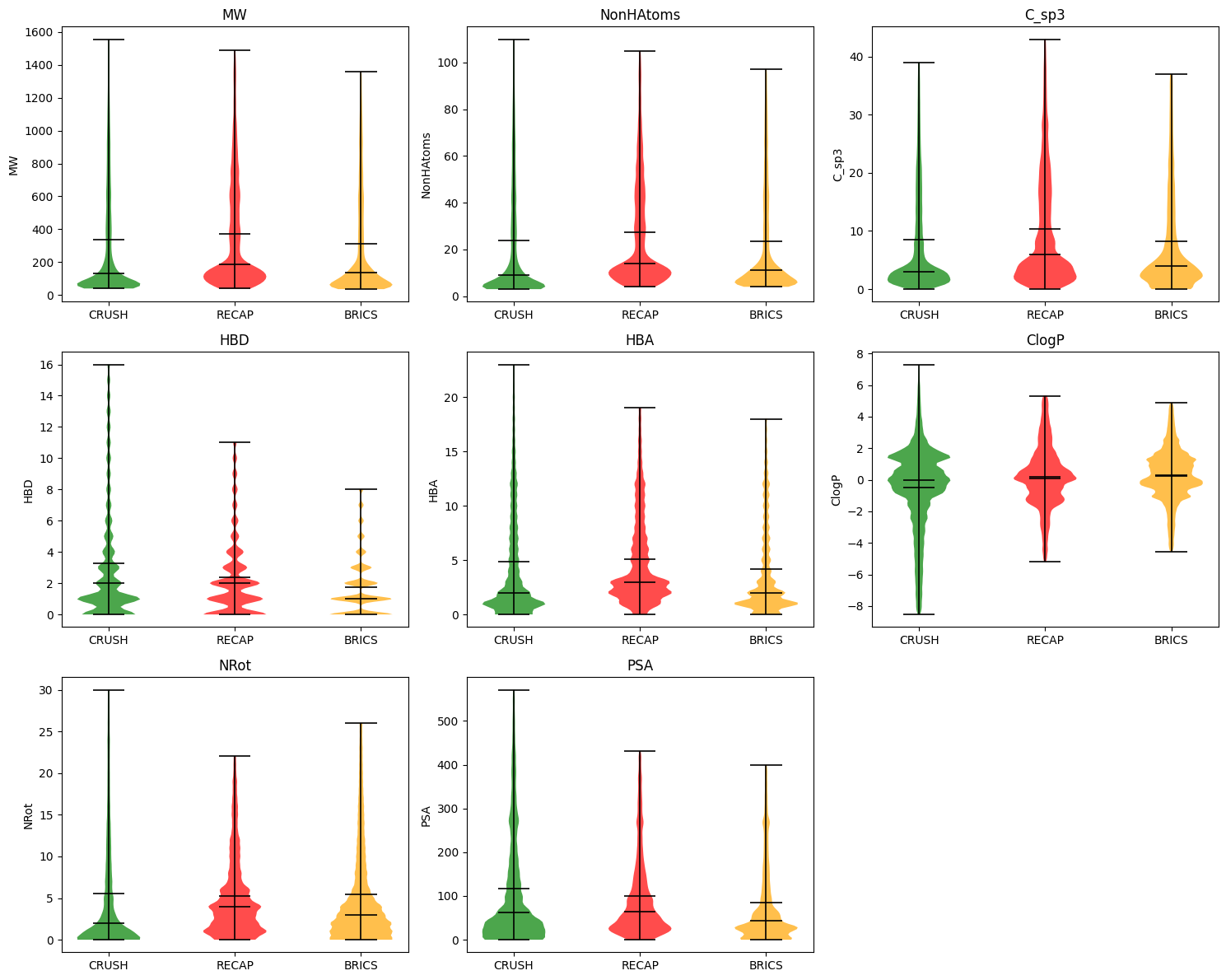


**Figure S9.** Comparison of fragments' properties generated from Macrocycle-DB.


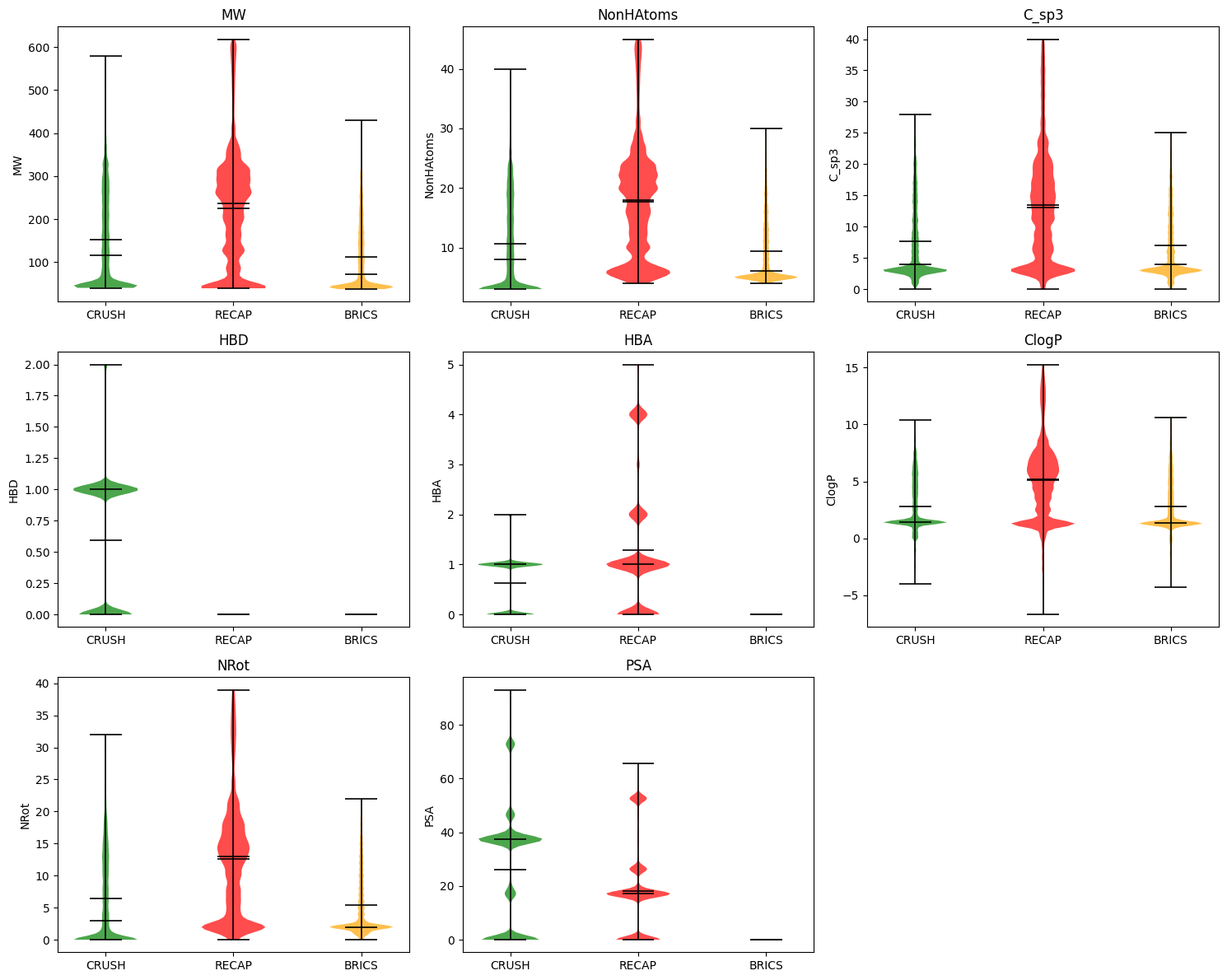


**Figure S10.** Comparison of fragments' properties generated from FooDB.
